# Wavelet event-related EEG phase coherence as a discriminant biomarker of the cognitive status in Parkinson’s and Lewy body disease

**DOI:** 10.3389/fnhum.2026.1696861

**Published:** 2026-04-02

**Authors:** Sümeyye Özdemir, Ayşenur Akan, Tuba Aktürk, Ebru Yıldırım, Nesrin Helvacı Yılmaz, Lütfü Hanoğlu, Claudio Babiloni, Bahar Güntekin

**Affiliations:** 1 Neuroscience Research Center, Research Institute for Health Sciences and Technologies (SABITA), Istanbul Medipol University, Istanbul, Türkiye; 2 Department of Neuroscience, Graduate School of Health Science, Istanbul Medipol University, Istanbul, Türkiye; 3 Department of Cognitive Neuroscience, Faculty of Psychology and Neuroscience, Maastricht University, Maastricht, Netherlands; 4 Neuroscience and Neurotechnology Center of Excellence (NÖROM), Gazi University, Ankara, Türkiye; 5 Department of Neurology, School of Medicine, Istanbul Medipol University, Istanbul, Türkiye; 6 Department of Physiology and Pharmacology “Vittorio Erspamer”, Sapienza University of Rome, Rome, Italy; 7 Hospital San Raffaele Cassino, Cassino, Italy; 8 Department of Biophysics, School of Medicine, Istanbul Medipol University, Istanbul, Türkiye

**Keywords:** dementia with Lewy bodies, EEG functional connectivity, machine learning classification, Parkinson’s disease dementia, phase coherence, visual oddball

## Abstract

**Background:**

Human cognition is derived from functional cortical long-range connectivity, as reflected by phase synchronization between electrode pairs of low-frequency electroencephalographic (EEG) activity <8 Hz related to cognitive tasks.

**Methods:**

We tested the hypothesis that such an EEG marker, combined with machine learning, can discriminate between Parkinson’s disease (PD) with mild cognitive impairment (MCI) and dementia (D) and those with dementia with Lewy bodies (DLB). Event-related EEG delta (1–3.5 Hz) and theta (4–7 Hz) phase coherence were computed from EEG activity recorded during a visual oddball task in healthy controls (HC, *N* = 24) and PD-MCI (*N* = 20), PDD (*N* = 18), and DLB (*N* = 11) patients.

**Results:**

Using delta-band coherence as input, the comparison between HC and PD-MCI yielded an AUC of approximately 0.79 and an accuracy of 86.4%. Higher discriminative performance was observed for HC versus PDD, reaching an AUC near 0.92 with an overall accuracy of 94.6%. In the classification of HC versus DLB participants, the model achieved 83.3% sensitivity and 88.9% specificity, with an AUC around 0.77. Theta-band models showed comparable results, with average AUC values of about 0.75 for HC vs. DLB and slightly above 0.80 for HC vs. PDD, while classification of HC vs. PD-MCI remained in a moderate range.

**Conclusion:**

These findings suggest that event-related EEG phase coherence at <8 Hz is a promising EEG correlate of cognitive deficits in patients with PDD and DLB, offering insights into disrupted network dynamics of cortical activity related to cognitive processes and potential biomarkers for testing new drugs for cognitive enhancement and disease monitoring.

## 1 Introduction

Neurodegenerative diseases constitute an increasing global health burden as life expectancy rises, particularly in developed countries. Dementia currently affects approximately 55 million individuals worldwide, with annual global costs surpassing USD 1.3 trillion. Parkinson’s disease (PD) is among the fastest growing neurodegenerative disorders, affecting an estimated 11.77 million individuals globally in 2021 (Li et al., 2025). Cognitive impairment and dementia represent major contributors to disability, reduced quality of life, institutionalization, and mortality in PD. Within the broader dementia spectrum, dementia with Lewy bodies (DLB), characterized by widespread α-synuclein pathology, accounts for approximately 5–8% of all dementia cases.

Despite the substantial clinical and societal impact, dementia in Parkinsonian and Lewy body spectrum disorders remains under-recognized (Alzheimer’s Association, 2025; Ratnavel et al., 2025), and early differentiation between cognitive phenotypes—such as PD with mild cognitive impairment (PD-MCI), Parkinson’s disease dementia (PDD), and DLB—remains challenging in routine clinical practice. Delayed or inaccurate phenotypic classification limits timely intervention, complicates clinical decision-making, and increases caregiver and health-care burden. These challenges highlight the need for accessible, objective, and scalable biomarkers that can be feasibly deployed in outpatient settings to support early diagnosis, phenotypic stratification, and longitudinal monitoring of disease progression and treatment response. Electroencephalography (EEG) is particularly well suited for clinical application, offering a direct and temporally precise assessment of large-scale neural dynamics with minimal patient burden and wide availability.

The human brain orchestrates large-scale information processing across distributed networks through the dynamic synchronization of neural oscillations (Babiloni et al., 2004; Srinivasan et al., 2007). During cognitive task engagement, this coordination creates transient temporal windows that facilitate neural signal integration and is reflected in phase synchronization between electroencephalographic (EEG) signals recorded at spatially distributed electrode pairs, particularly in low-frequency bands (<8 Hz). Event-related (ER) delta (1–3.5/4 Hz) and theta (4–7 Hz) oscillations play a critical role in attention, memory, and executive functions by supporting long-range interregional communication (Başar et al., 1999; Klimesch, 1999). Accordingly, EEG phase coherence was selected as the primary analytic marker in the present study, as it directly quantifies coordination between distributed brain regions and provides interpretable signatures of large-scale neural communication that extend beyond local signal amplitude alone (Haufe et al., 2014).

In neurodegenerative disorders, cognitive decline is increasingly conceptualized as a *disconnection syndrome*, in which progressive disruption of large-scale network communication underlies cognitive dysfunction. Consistent with this framework, growing evidence indicates that disruptions in delta and theta phase synchronization may reflect cortical functional dysconnectivity underlying cognitive decline in neurodegenerative diseases such as PD at the stage of PD-MCI, PDD, and DLB (Güntekin and Başar, 2016; Yener et al., 2019). For example Güntekin et al. (2019) reported graded reductions in theta inter-trial phase coherence and spectral power during auditory and visual oddball target processing, with the lowest values in PDD, intermediate reductions in PD-MCI, and highest values in cognitively unimpaired PD, most prominently at frontal–central electrode sites with a right-hemisphere predominance.

In another study, Fide et al. (2023) reported reduced delta, theta, and alpha intrahemispheric and midline coherence during visual oddball target processing in patients with Alzheimer’s disease (AD) relative to healthy controls (HC), whereas individuals with amnestic mild cognitive impairment (aMCI) exhibited increased coherence, interpreted as reflecting compensatory or potentially maladaptive network reorganization at an early disease stage. Furthermore, Hünerli-Gündüz et al. (2023) reported reduced delta power and phase-locking values (PLVs) in patients with PD-MCI during an auditory oddball paradigm, and proposed that these reductions are associated with structural atrophy of the thalamus, putamen, and hippocampus, as assessed by magnetic resonance imaging (MRI). In agreement, Güntekin et al. (2021), also found that both delta and theta PLV were significantly reduced during auditory oddball paradigm in patients with PD-D, Alzheimer’s disease with dementia (ADD), DLB, and vascular cognitive impairment (VCI) compared to HC.

Although prior findings are informative, they remain insufficient for clinical assessment of cognitive deficits in PD and DLB. To address this gap, we implemented a structured and interpretable machine-learning pipeline designed for both interpretability and predictive reliability. Phase-locking values (PLVs) in the delta and theta bands were extracted as connectivity features (Ruiz-Gómez et al., 2019; Cao et al., 2025), and Bolasso—a bootstrapped extension of LASSO combining bootstrap resampling with L1 regularization (Bach, 2008; Yang et al., 2023; Tibshirani, 1996)—was applied to enhance feature stability and limit overfitting in high-dimensional EEG spaces with modest sample sizes. Regularized linear models impose sparsity, improve generalization, and enable interpretable feature selection in neural data (Abraham et al., 2014); accordingly, LASSO-based logistic regression provides a robust framework for EEG-based neurological classification, with recent clinical EEG studies demonstrating its predictive utility (Mao et al., 2025). Finally, supervised classification analyses (e.g., HC vs. PDD) were performed using 10-fold cross-validation and regularization to ensure robust and generalizable performance. Additionally, our approach aligns with recent trends favoring explainable and lightweight EEG models, such as xEEGNet (Zanola et al., 2025).

To our knowledge, no prior study has combined wavelet-based EEG phase-connectivity metrics with ML classifiers to differentiate healthy controls, PD-MCI, PDD, and DLB within a single task-based analytic framework. The present work integrates task performance (oddball error scores) and global cognitive status (MMSE) alongside edge- and network-level EEG features, enabling coherence alterations to be interpreted in relation to both behavioral performance and clinical cognitive status within a rigorously defined and diagnostically homogeneous cohort. This multimodal and internally consistent framework strengthens biological interpretability and distinguishes the present study from prior oddball or EEG connectivity investigations, which have typically examined Parkinsonian and Lewy body disorders separately or focused on a single analytic level. At the same time, translation of EEG connectivity measures into clinically meaningful outcomes remains limited. Existing findings in Lewy body– and Parkinsonian-spectrum disorders are difficult to compare due to substantial heterogeneity (di Biase et al., 2023; Kucikova et al., 2024; Law et al., 2020; van der Zande et al., 2018) in acquisition protocols and analysis pipelines, and connectivity metrics remain less consistently standardized and validated than traditional spectral EEG markers in comparative studies. Against this background, the present study provides a systematic and integrative step toward bridging EEG connectivity measures with clinically relevant cognitive phenotypes.

To address this gap, the present study specifically tested the hypothesis that:

(1) wavelet-based event-related delta and theta phase coherence between electrode pairs would progressively decrease with increasing cognitive impairment across PD-MCI, PDD, and DLB relative to HC, with the most pronounced disruptions observed in dementia-stage patients; and
(2) these connectivity measures would support individual-level classification, yielding higher discriminative accuracy for HC vs. PDD and HC vs. DLB comparisons than for HC vs. PD-MCI.

## 2 Materials and methods

### 2.1 Participants

#### 2.1.1 Recruitment

Demographic characteristics for all groups are summarized in Table 1. Groups did not differ significantly in age or years of education (ANOVA, *p* > 0.05). Mean age (±SD) was 61.3 ± 7.95 years for HC, 67.5 ± 8.59 for PD-MCI, 70.2 ± 7.41 for PDD, and 70.3 ± 9.27 for DLB participants. Mean years of education ranged from 6.7 to 10.0 years across groups.

**Table 1:** Demographics across groups.

Variable	HC	PD-MCI	PDD	DLB
*N* (Total = 73)	24	20	18	11
Sex (M: F)	13:11	15:5	17:1	8:3
Age (Mean ± SD)	61.3 ± 7.95	67.5 ± 8.59	70.2 ± 7.41	70.3 ± 9.27
Education (Mean ± SD)	10.0 ± 4.42	6.70 ± 2.39	7.0 ± 3.66	7.55 ± 3.80

*No significant differences were found across groups for age or education (ANOVA, *p* > 0.05). Age was included as a covariate in secondary analyses to mitigate potential confounding.

Disease duration was not consistently available across all participants and was therefore not included as a descriptive or analytical variable.

A total of 73 participants (aged 50–80) were recruited from the Neurology Outpatient Clinic of Istanbul Medipol University Hospital and assigned to four groups: PD-MCI (*N* = 20, 15 male), PDD (*N* = 18, 17 male), DLB (*N* = 11, 8 male), and age-matched healthy controls (*N* = 24, 13 male). All of our patients were on dopaminergic medication; they had their daily medication just 1 h before the EEG recordings. They had normal or corrected vision, no reported hearing impairments, and were naive to the task. The study was approved by the Istanbul Medipol University Ethics Committee (Approval No: 10840098-51, E30217-E30218) and was conducted in accordance with the Declaration of Helsinki. Informed consent was obtained from all participants or their caregivers before the study commenced. Participants did not receive financial compensation for their participation.

#### 2.1.2 Inclusion and exclusion criteria

PDD diagnosis followed Movement Disorder Society criteria (Emre et al., 2007) and UK Brain Bank guidelines (Georgiev et al., 2015), with MMSE < 26 (Güngen et al., 2002), CDR ≥ 0.5, and functional impairment ≥1.5 SD below norms (Huebl et al., 2014). PD-MCI was defined by ≥1.5 SD cognitive impairment in ≥2 tests within one domain. DLB diagnosis followed McKeith et al. (2005) criteria, with MMSE < 26 and functional impairment ≥1.5 SD. Healthy controls scored ≥26 on the MMSE, no neurological/psychiatric history, and no chronic neuroactive medication use. Exclusion criteria included other dementias, cognition-altering drugs, substance abuse, stroke, TBI, epilepsy, psychiatric/neurological conditions, GDS > 13, unstable medical conditions, and MRI abnormalities. Groups were age-matched with no significant differences (*p* > 0.05) (Podcasy and Epperson, 2016; Walker et al., 2015).

### 2.2 Task design and procedure

**Figure 1:**
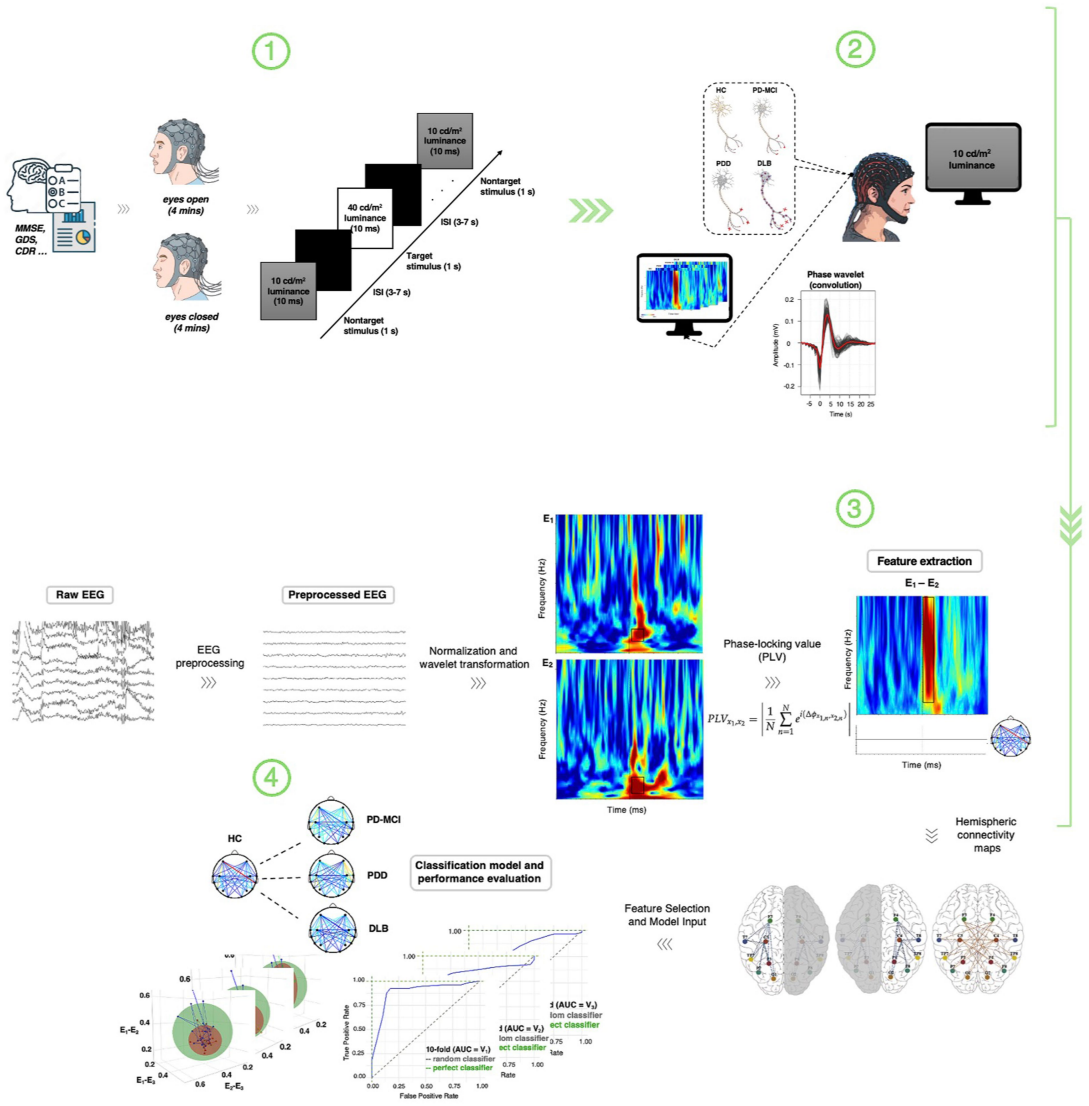
End-to-end workflow of the study. Overview of the experimental and analytical pipeline organized into four stages: (1) Clinical and neuropsychological assessment, baseline EEG recording, and visual oddball task structure and stimulus timeline; (2) task-based EEG acquisition during target and non-target processing; (3) EEG preprocessing, wavelet-based time–frequency decomposition, and phase-locking value (PLV) connectivity estimation across inter- and intrahemispheric electrode pairs; and (4) multivariate feature selection, network characterization, low-dimensional embedding, and regularized classification with cross-validated and permutation-based performance evaluation. The numbered layout highlights the integrative and hypothesis-driven nature of the pipeline, spanning task-evoked neurophysiology, network analysis, and predictive modeling across Parkinsonian dementia subtypes.

Each participant completed three parts in a single session (Figure 1): (1) neuropsychological evaluation, (2) resting-state EEG, and (3) task-based EEG during a visual oddball paradigm.

#### 2.2.1 Neuropsychological evaluation

Cognitive assessment included the Turkish MMSE (max score = 30) (Güngen et al., 2002) to assess global cognition; the Öktem Verbal Memory Processes Test (OVMPT) for verbal memory (Tanör, 2011); semantic and phonemic Verbal Fluency Tests (Crawford et al., 2018); the Clinical Dementia Rating Scale (CDR) for dementia severity (Morris, 1993, 1997); and the 30-item Geriatric Depression Scale (GDS) for mood screening (Yesavage et al., 1982).

#### 2.2.2 Task procedure

The task was implemented in E-Prime (Psychology Software Tools Inc., Pittsburgh, PA) to assess neural responses to target and nontarget stimuli. Stimuli were presented at two luminance levels—10 cd/m^2^ (nontarget) and 40 cd/m^2^ (target)—on a 22-inch monitor (75 Hz, 10 ms rise time, 1,000 ms duration). Each trial consisted of 120 stimuli included 40 targets (33.3%) and 80 nontargets (66.6%) in randomized order that was independently generated for each participant, and lasted approximately 12 min, with a variable interstimulus interval of 3–7 s to reduce anticipatory effects. Participants were seated approximately 100 cm from the monitor at eye level in a dimly lit, sound-attenuated room to ensure visual comfort and stable viewing conditions. They were instructed to silently count the target stimuli while ignoring nontargets and to report the total number of targets post-trial. A sample session confirmed task comprehension. Behavioral performance was quantified as the absolute counting error relative to the correct target number (i.e., |40 – response|).

A schematic overview of the task structure and stimulus timeline is provided in Figure 1, illustrating the luminance-defined stimuli, stimulus duration, and interstimulus interval.

### 2.3 EEG acquisition and preprocessing

#### 2.3.1 Recording

EEG signals were recorded using a 32-channel BrainCap with Multitrodes (EasyCap GmbH, Germany), with electrodes placed according to the international 10–20 system. Electrode sites included: Fp1, Fp2, F7, F3, Fz, F4, F8, FT7, FC3, FCz, FC4, FT8, Cz, C3, C4, T7, T8, TP7, CP3, CPz, CP4, TP8, P3, Pz, P4, P7, P8, O1, Oz, and O2. Linked Ag/AgCl electrodes on the earlobes (A1 + A2) served as references, and EOG was recorded from the medial upper and lateral orbital rim of the left eye. Electrode impedance was kept below 10 kΩ. Signals were amplified using a BrainAmp MR Plus 32-channel DC system (Brain Products GmbH, Germany), bandpass filtered between 0.01–250 Hz, and sampled at 500 Hz. Recordings were performed in a dimly lit, shielded room to minimize external interference.

#### 2.3.2 Preprocessing

Event-related EEG data were preprocessed in BrainVision Analyzer (BVA). Signals were downsampled to 256 Hz, band-pass filtered (0.01–60 Hz), and re-referenced to linked mastoids. Ocular artifacts were removed using independent component analysis (ICA). Data were segmented into 6 s epochs (−3 to +3 s) for delta and 2 s epochs (−1 to +1 s) for theta, separately for target and non-target trials. Manual artifact rejection was performed to ensure clean data for time-frequency and connectivity analyses.

After ICA and manual artifact rejection, an average of 17.6 ± 2.9 (HC), 15.1 ± 2.5 (PDD), 14.9 ± 1.8 (PD-MCI), and 14.3 ± 2.3 (DLB) target epochs, as well as 19.7 ± 3.2 (HC), 16.5 ± 2.7 (PDD), 18.6 ± 2.8 (PD-MCI), and 16.5 ± 2.6 (DLB) non-target epochs per participant, were retained for subsequent connectivity analyses.

### 2.4 EEG analysis

#### 2.4.1 Event-related EEG

Time-frequency analyses were conducted using the Gabor-normalized Complex Morlet Wavelet Transform (WT). A fixed wavelet width of three cycles was applied at each center frequency, yielding a temporal standard deviation of *σ*ₜ = 3 / (2πf) and a corresponding frequency-domain standard deviation of σ𝑓 = f / 3 across 30 logarithmically spaced bins for delta (1–3.5 Hz) and theta (4–7 Hz) bands. Prior to WT, Current Source Density (CSD) transformation was applied to reduce volume conduction (Spline order: 4; Legendre degree: 10; Lambda: 1e–5) (Thatcher et al., 1986). Phase-based connectivity was quantified using PLV, calculated from WT phase outputs, separately for target and non-target conditions without baseline normalization. As defined by Lachaux et al. (1999, 2002), with implementation consistent with contemporary time–frequency approaches (Cohen, 2014). Instantaneous phase estimates were obtained from the complex Morlet wavelet transform (Addison, 2002; Bostanov, 2004), and PLV (PLV; range 0–1) was computed for each electrode pair as the magnitude of the average unit-length phase difference vectors across trials, quantifying functional connectivity between channels. Higher PLV indicates more stable phase relationships across trials. This metric is relatively robust to amplitude fluctuations and volume-conduction artifacts when combined with spatial filtering (i.e., CSD). Delta-band PLV was averaged (E1) over 0–600 ms, and theta over 0–300 ms post-stimulus, across all electrode pairs. Outliers were excluded using the IQR method (values beyond Q1–3*IQR or Q3 + 3*IQR), applied separately to coherence, neuropsychological, and behavioral data.

E1 (Phase Locking Value)

$$\mathrm{PLV}(f,t)=|\frac{1}{N}\sum_{\text{trial}=1}^{N}\exp(j(\phi_{y,\text{trial}}(f,t)-\phi_{x,\text{trial}}(f,t)))|$$

where *φₓ*, trial(*f*,*t*) and *φᵧ*, trial(*f*,*t*) are the instantaneous phase values of two signals. The PLV was calculated separately for the delta (1–3.5 Hz) and theta (4–7 Hz) bands.

### 2.5 Statistical analysis

All statistical and machine learning analyses were conducted in R version 4.2.1 (2022-06-23).

#### 2.5.1 Behavioral and neuropsychological analysis

Task performance was measured by error scores—incorrect responses to target stimuli—reflecting engagement. Although a full neuropsychological battery was administered, MMSE was used as the primary cognitive measure due to its clinical relevance, validation across dementia subtypes, and consistency across groups (Kamarajan and Porjesz, 2015; Rossini et al., 2006; Handayani et al., 2018). Linear regression models were run per group to examine associations between MMSE, error scores, and coherence. To assess whether coherence–behavior relationships were independent of demographic confounds, additional multiple regression models were performed including age as a covariate. Subject-level weighted average coherence during target trials served as single coherence predictor per participant. *p*-values were computed and corrected for multiple comparisons using the FDR. Standardized regression coefficients (β) reflect effect sizes derived from models fit at the electrode-pair level (Table 2).

**Table 2:** Statistics for the delta and theta band across groups [mean (SD)] and hemispheres, and sensitivity analysis.

Measure	HC	PD-MCI	PDD	DLB	ANOVA-statistics	*Post-hoc* comparisons	Sensitivity analysis [CI (lower, upper)]
Delta phase values							
Interhemispheric	0.322 (0.067)	0.310 (0.042)	0.282 (0.040)	0.292 (0.032)	*F* = 2.283, *p* = 0.087, η^2^*p* = 0.090	HC–PD-MCI (p = 0.048*, g = 26)	HC–PD-MCI [CI (−0.016, 0.044), *p* = 0.038*]
Left hemisphere	0.316 (0.060)	0.322 (0.042)	0.303 (0.041)	0.309 (0.028)		HC–PDD (*p* = 0.035*, *g* = 0.73)	HC–PDD [CI (0.007, 0.067), *p* = 0.029*]
Right hemisphere	0.346 (0.057)	0.313 (0.041)	0.282 (0.032)	0.292 (0.022)		HC–DLB (*p* = 0.049*, *g* = 0.54)	HC–DLB [CI (−0.005, 0.055), *p* = 0.032*]
MMSE					Regression-statistics	Within-group descriptives	
Interhemispheric	0.358 (0.159)	0.322 (0.076)	0.315 (0.082)	0.298 (0.076)	*R*^2^ = 0.395, β = 0.061, *p* < 0.01*	HC (*R*^2^ = 0.178), PD-MCI (*R*^2^ = 0.23), PDD (*R*^2^ = 0.56*), DLB (*R*^2^ = 0.192)	PD-MCI [−5.54, −4.56], PDD [−7.31, −6.30], DLB [−7.17, −5.97]
Intrahemispheric	0.409 (0.139)	0.329 (0.093)	0.299 (0.080)	0.332 (0.075)	*R*^2^ = 0.425, β = 0.088, *p* < 0.001*	HC (*R*^2^ = 0.101), PD-MCI (*R*^2^ = 0.168), PDD (*R*^2^ = 0.47*), DLB (*R*^2^ = 0.35*)	PD-MCI [−5.46, −4.48], PDD [−7.32, −6.32], DLB [−7.08, −5.88]
Error scores							
Interhemispheric	0.359 (0.132)	0.313 (0.075)	0.299 (0.096)	0.319 (0.080)	*R*^2^ = 0.56, β = −0.080, *p* < 0.001*	HC (*R*^2^ = 0.198), PD-MCI (*R*^2^ = 0.275*), PDD (*R*^2^ = 0.495*), DLB (*R*^2^ = 0.315*)	PD-MCI [1.63, 2.39], PDD [6.11, 6.88], DLB [7.44, 8.36]
Intrahemispheric	0.371 (0.115)	0.343 (0.102)	0.310 (0.086)	0.306 (0.066)	*R*^2^ = 0.68, β = −0.155, *p* < 0.001*	HC (*R*^2^ = 0.133), PD-MCI (*R*^2^ = 0.330*), PDD (*R*^2^ = 0.670*), DLB (*R*^2^ = 0.298*)	PD-MCI [1.61, 2.36], PDD [6.12, 6.90], DLB [7.44, 8.36]
Theta phase values							
Interhemispheric	0.292 (0.062)	0.269 (0.051)	0.288 (0.045)	0.279 (0.029)	*F* = 1.188, *p* = 0.321, η^2^*p* = 0.040	HC–PD-MCI (*p* > 0.05, *g* = 0.22)	HC–PD-MCI [CI (−0.006, 0.041), *p* = 0.048*]
Left hemisphere	0.295 (0.055)	0.301 (0.049)	0.282 (0.042)	0.308 (0.032)		HC–PDD (*p* = 0.042*, *g* = 0.43)	HC–PDD [CI (−0.004, 0.043), *p* = 0.029*]
Right hemisphere	0.292 (0.052)	0.269 (0.051)	0.288 (0.045)	0.279 (0.028)		HC–DLB (*p* = 0.048*, *g* = 0.30)	HC–DLB [CI (−0.045, 0.038), *p* = 0.032*]
MMSE					Regression-statistics	Within-group descriptives	
Interhemispheric	0.324 (0.116)	0.275 (0.052)	0.280 (0.070)	0.310 (0.083)	*R*^2^ = 0.289, β = 0.057, *p* < 0.05*	HC (*R*^2^ = 0.25), PD-MCI (*R*^2^ = 0.32*), PDD (*R*^2^ = 0.44*), DLB (*R*^2^ = 0.23)	PD-MCI [−4.56, −3.51], PDD [−6.34, −5.26], DLB [−6.35, −5.07]
Intrahemispheric	0.350 (0.120)	0.310 (0.14)	0.290 (0.12)	0.310 (0.11)	*R*^2^ = 0.408, β = 0.068, *p* < 0.01*	HC (*R*^2^ = 0.20), PD-MCI (*R*^2^ = 0.37*), PDD (*R*^2^ = 0.43*), DLB (*R*^2^ = 0.30*)	PD-MCI [−5.46, −4.49], PDD [−7.32, −6.32], DLB [−7.12, −5.92]
Error scores							
Interhemispheric	0.290 (0.10)	0.140 (0.27)	0.129 (0.080)	0.280 (0.07)	*R*^2^ = 0.44, β = −0.078, *p* < 0.05*	HC (*R*^2^ = 0.18), PD-MCI (*R*^2^ = 0.38*), PDD (*R*^2^ = 0.42*), DLB (*R*^2^ = 0.34*)	PD-MCI [1.63, 2.38], PDD [6.11, 6.88], DLB [7.37, 8.29]
Intrahemispheric	0.310 (0.06)	0.290 (0.07)	0.310 (0.06)	0.290 (0.07)	*R*^2^ = 0.56, β = −0.86, *p* < 0.01*	HC (*R*^2^ = 0.19), PD-MCI (*R*^2^ = 0.33*), PDD (*R*^2^ = 0.44*), DLB (*R*^2^ = 0.32*)	PD-MCI [1.61, 2.36], PDD [6.12, 6.90], DLB [7.44, 8.36]

The omnibus ANOVA for delta and theta did not reach significance at the conventional threshold [*F*(3, 69) = 2.283, *p* = 0.087; *F*(3, 69) = 1.188, *p* = 0.321]. Nevertheless, given our a priori expectation of reduced connectivity in patient groups relative to HC, we conducted planned pairwise contrasts (*p*-values FDR-corrected). These results are consistent with our a priori hypothesis; however, as the omnibus tests were not significant, they should be interpreted cautiously as exploratory. Effect sizes for planned post-hoc contrasts are reported as bias-corrected standardized mean differences (Hedges’ g) computed from model-estimated marginal means (EMMs).

CI = 95% confidence interval (regression coefficients).

#### 2.5.2 Signal analysis

A mixed-design repeated measures ANOVA was conducted to assess differences in EEG coherence across groups (HC, PDD, PD-MCI, DLB), with Group as a between-subjects factor, and Electrode Pair (40 pairs), Frequency Band (Delta, Theta), Stimulus Type (Target, Nontarget), and Hemisphere (Left, Right, Inter) as within-subjects factors (R package: *lme4*). The 40 electrode pairs included: F3-T7, F3-T8, F4-T7, F4-T8, F3-P3, F3-P4, F4-P3, F4-P4, F3-TP7, F3-TP8, F4-TP7, F4-TP8, F3-P7, F3-P8, F4-P7, F4-P8, F3-O1, F3-O2, F4-O1, F4-O2, C3-T7, C3-T8, C4-T7, C4-T8, C3-P3, C3-P4, C4-P3, C4-P4, C3-TP7, C3-TP8, C4-TP7, C4-TP8, C3-P7, C3-P8, C4-P7, C4-P8, C3-O1, C3-O2, C4-O1, C4-O2. Post-hoc pairwise comparisons were FDR-corrected (*p* < 0.05), and Greenhouse–Geisser corrections were applied for sphericity violations. Effect sizes for planned post-hoc contrasts are reported as bias-corrected standardized mean differences (Hedges’ g) computed from model-estimated marginal means (EMMs).

To limit the multiple-comparison burden inherent in EEG connectivity analyses, PLV estimates were restricted to a predefined set of 40 electrode pairs selected *a priori* based on their relevance to large-scale cognitive networks (e.g., fronto–temporal, fronto–parietal, centro–parietal, and temporo–occipital connections). This hypothesis-driven selection substantially reduced dimensionality and avoided mass-univariate testing across thousands of sensor pairs, which can severely reduce statistical power when naïve corrections are applied (Li et al., 2025).

PLV was analyzed at multiple, explicitly defined aggregation levels, depending on the statistical objective. At the edge level, PLV was computed separately for each of the 40 predefined electrode pairs, and these pairwise values were used directly in electrode-pair–level analyses, including bootstrap/permutation testing, feature selection, and supervised classification. At the hemisphere level, PLV values were aggregated within participants by averaging across all eligible electrode pairs belonging to a given category (left intrahemispheric, right intrahemispheric, or interhemispheric), yielding a single summary coherence value per category, frequency band, and participant. These aggregated measures were used in mixed-design ANOVA, regression analyses linking coherence to behavioral and neuropsychological measures, and descriptive group comparisons. At the network level, weighted adjacency matrices constructed from pairwise PLV values were used to compute graph-theoretic metrics (global efficiency, local efficiency, and small-worldness) without prior averaging, preserving the topological structure of functional connectivity. This multi-level strategy allowed us to combine fine-grained edge-level sensitivity with interpretable hemisphere- and network-level summaries while maintaining statistical consistency across analyses.

### 2.6 Functional network characterization

To assess group-level separability, we constructed a 3D *coherence feature embedding* using the most discriminative electrode pairs (delta and theta bands) selected via LASSO or regression weighting. Each participant was projected into this space, with axes representing coherence values for the selected pairs. Group distributions were visualized as mean-centered ellipsoids with standard deviation contours (*rgl* package). A multivariate linear model assessed separability (β coefficients, *p* < 0.05), serving as a summary view of coherence-based disruption across groups. Feature selection and embedding were performed within the same dataset and were intended for exploratory characterization of coherence. The use of a low-dimensional embedding, fixed-density network construction, and group-level statistical testing was intended to limit model complexity and reduce overfitting in the context of a modest sample size. *Network efficiency* was evaluated using graph-theoretic metrics implemented in the *igraph* package. Local efficiency (LE) which measures the efficiency of information transfer within the immediate neighborhood of each node and reflects functional segregation and the resilience of local subnetworks and global efficiency (GE) which quantifies the capacity of the functional network to integrate information across distant nodes were computed from weighted, undirected phase coherence networks in the delta and theta frequency bands. Functional connectivity matrices were thresholded at 10% density (90% sparsity) to retain the strongest connections. Subject-specific graphs were constructed to preserve the physiological structure of coherence-based connectivity. In addition, clustering coefficient (C) and characteristic path length (L) were normalized against 100 degree-matched random networks per subject, and small-worldness (*σ*) was used to characterize the balance between network integration and segregation and defined as (C/C_rand) / (L/L_rand). Efficiency values are reported as raw measures under fixed edge density, whereas small-worldness values reflect normalized topological characteristics relative to random networks.

These analyses were designed to highlight discriminative coherence patterns, providing a data-driven foundation for subsequent classification.

E2 (Global Efficiency)

$$E_{\text{global}}=\frac{1}{N}\sum_{i\neq j}\frac{1}{d_{\mathrm{ij}}}$$

where *N* is the number of nodes and d*_ij_* denotes the shortest path length between nodes *i* and *j*. Higher global efficiency reflects more efficient long-range information transfer.

E3 (Local Efficiency)

$$E_{\mathrm{loc}}=\frac{1}{N}\sum_{i}\frac{1}{|V_{i}|(|V_{i}|-1)}\sum_{j,k\in V_{i}}\frac{1}{d_{\mathrm{jk}}}$$

where *V_i_* is the set of neighbors of node *i*, and *d_jk_* is the shortest path length between nodes *j* and *k* within the subgraph induced by V*_i_*.

E4 (Small-world Coefficient)

$$\sigma =\frac{C/C_{\mathrm{rand}}}{L/L_{\mathrm{rand}}}$$

where *C* and *L* are the clustering coefficient and the characteristic path length of the observed network, and *C_rand_*, *L_rand_* are the same measures from a random network. A higher *σ* suggests an optimal network organization that balances segregation and integration.

Global efficiency, local efficiency, and small-worldness were prioritized because they provide complementary and interpretable indices of network integration, segregation, and their balance—core organizational principles of brain networks widely applied in EEG connectivity studies of neurodegeneration (Vecchio et al., 2017; Franciotti et al., 2019; Jalili, 2016; Vecchio et al., 2024). Accordingly, the present study employed fixed-density thresholding and focused on global and local efficiency metrics to enhance comparability across participants and to emphasize stable, network-level markers of cognitive dysfunction.

### 2.7 Supervised classification of dementia subtypes and performance evaluation

To classify clinical groups, we applied LASSO logistic regression with 10-fold cross-validation (package: *glmnet*), with the regularization parameter (λ) selected to minimize binomial deviance along the LASSO regularization path enabling simultaneous feature selection and model evaluation. Two standard solutions were retained for evaluation: the model corresponding to the minimum cross-validated deviance (λ_min) and a more conservative solution defined by the one-standard-error criterion (λ_1se). These complementary solutions were used to assess predictive performance as well as model parsimony and stability. In each training fold, the most predictive EEG phase coherence features (delta/theta-band PLV values) were identified by shrinking non-informative coefficients to zero via L1 regularization. For each frequency band (delta and theta), models were constructed using a fixed candidate feature space comprising *p* = 40 electrode-pair coherence predictors, defined *a priori* and applied consistently across all group comparisons.

Each observation corresponded to a single participant, ensuring that no participant appeared in more than one fold. Predictor variables were standardized within the modeling procedure using glmnet’s default setting (standardize = TRUE), and regularization parameter selection (λ_min and λ_1se) was performed entirely within the cross-validation framework.

For classification, the dependent variable (DV) was binary diagnostic group membership (HC vs. PD-MCI, HC vs. PDD, or HC vs. DLB), while independent variables (IVs) consisted of participant-level EEG phase coherence features (PLV values) computed for predefined electrode pairs in the delta (1–3.5 Hz) and theta (4–7 Hz) frequency bands.

This ensured that feature selection was performed strictly within the training data of each fold, preventing information leakage from the test set and yielding unbiased performance estimates. The resulting sparse models enhance both generalizability and interpretability of the classification (Bostanov, 2004; Rosset and Zhu, 2006). Regularization strength (λ) was optimized using both the minimum deviance and the 1-SE criteria (E5). The number of retained predictors (K) was defined as the number of non-zero coefficients in the refitted LASSO model at λ_min and λ_1se. Binary classification models were trained separately for each group comparison (HC vs. PDD, HC vs. PD-MCI, HC vs. DLB) and for delta and theta frequency bands. To improve feature selection stability, we additionally applied Bolasso (bootstrap-enhanced LASSO), retaining features selected in ≥85% of bootstrap iterations—a strategy shown to improve reproducibility in EEG-based prediction of cognitive status (Tibshirani, 1996; Fawcett, 2006).

E5

$$\hat{\beta }=\arg\min_{\beta }\frac{1}{n}\sum_{i=1}^{n}\rho_{i}(x_{i},y_{i})+\lambda {|\beta |}_{1}$$

where *λ* is a regularization parameter controlling feature selection. Top features were selected for classification.

Notably, several features consistently appeared across LASSO selections, and were also identified in earlier time–frequency and network-level analyses, supporting their convergence across analytical domains.

Classification performance was assessed via ROC curves generated from LASSO-selected features (package: *pROC*), with AUC values (Tibshirani, 1996) reported as threshold-independent accuracy metrics (E6). AUC values were computed for top-ranked LASSO features, with confidence intervals estimated via bootstrapping. Sensitivity and specificity were derived from confusion matrices based on cross-validated predictions. Unless otherwise stated, performance metrics (AUC, sensitivity, specificity) are reported for models selected at λ_min, while λ_1se is reported to characterize sparsity and robustness under stricter regularization.

To facilitate transparent interpretation of model complexity, the number of non-zero predictors retained by LASSO (K) was explicitly reported for both the λ_min and λ_1se solutions. Model sparsity was described using the following terminology: an *intercept-only* solution (*K* = 0), a *single-feature solution* (*K* = 1), a *sparse multivariate set* (*K* = 2–5), and a *compact multivariate set* (*K* ≥ 6). This descriptive framework reflects increasing model complexity while avoiding arbitrary thresholds for statistical significance.

E6

$$\mathrm{AUC}=\int_{0}^{1}\mathrm{TPR}(\mathrm{FPR})\;d(\mathrm{FPR})$$

where TPR (True Positive Rate) and FPR (False Positive Rate) were calculated from confusion matrices.

Classification outcomes were further quantified using confusion matrices (package: *caret*), reporting sensitivity, specificity, and accuracy based on true/false positive rates (Powers, 2011). Binary classifiers were pre-specified (one-vs-HC) to preserve interpretability and mitigate class-imbalance effects (Fernández et al., 2019); performance was summarized primarily using AUC with class-wise sensitivity and specificity. Analyses were conducted separately for delta and theta bands using PLV-based coherence features (Lachaux et al., 1999).

### 2.8 Sensitivity analysis and validation

#### 2.8.1 Data quality assessment

EEG quality was verified using variance metrics, IQR-based outlier detection, and a signal-to-noise ratio threshold (>15 dB). An eight-minute resting-state EEG (4 min eyes open/closed) was recorded pre-task to screen for epileptiform activity, abnormal slow waves, hemispheric asymmetries, and artifacts (e.g., muscle or eye movements).

#### 2.8.2 Bootstrapping and permutation testing

Model stability and coherence metrics were evaluated using 5,000-iteration bootstrapping (package: *boot*), applying the bias-corrected and accelerated (BCa) method to account for skew and bias—optimal for small, non-normal EEG datasets. For each iteration, coherence differences were recalculated on resampled data to construct BCa confidence intervals. To validate findings, permutation testing (package: *permute*, 5,000 iterations) was applied to the top-ranked coherence pairs (Lachaux et al., 1999), generating null distributions (Efron and Tibshirani, 1994) by shuffling group labels (E7). Empirical *p*-values were derived and corrected for multiple comparisons using the false discovery rate (FDR) method (Fisher, 1936).

E7

$$p=\frac{1}{M}\sum_{i=1}^{M}\theta_{i}\geq \Theta$$

where Θ represents the observed test statistic.

## 3 Results

We investigated event-related EEG delta- and theta-band phase coherence across 40 electrode pairs (20 intra- and 20 interhemispheric) during a visual oddball paradigm in the HC, PD-MCI, PDD, and DLB groups. To evaluate the potential of stimulus-locked phase synchronization as a clinical biomarker, we combined wavelet-based time-frequency analyses with behavioral performance (error rates in the oddball task) and scores on the MMSE, which reflect global cognitive status. A multistep pipeline was applied, including coherence feature extraction from EEG activity, 3D embedding, network efficiency estimation, and ML classification, to identify key discriminative interrelatedness of EEG delta- and theta-band responses to oddball targets between experimental and control groups (Figure 1).

### 3.1 Phase coherence alterations from EEG activity across dementia subtypes

**Figure 2:**
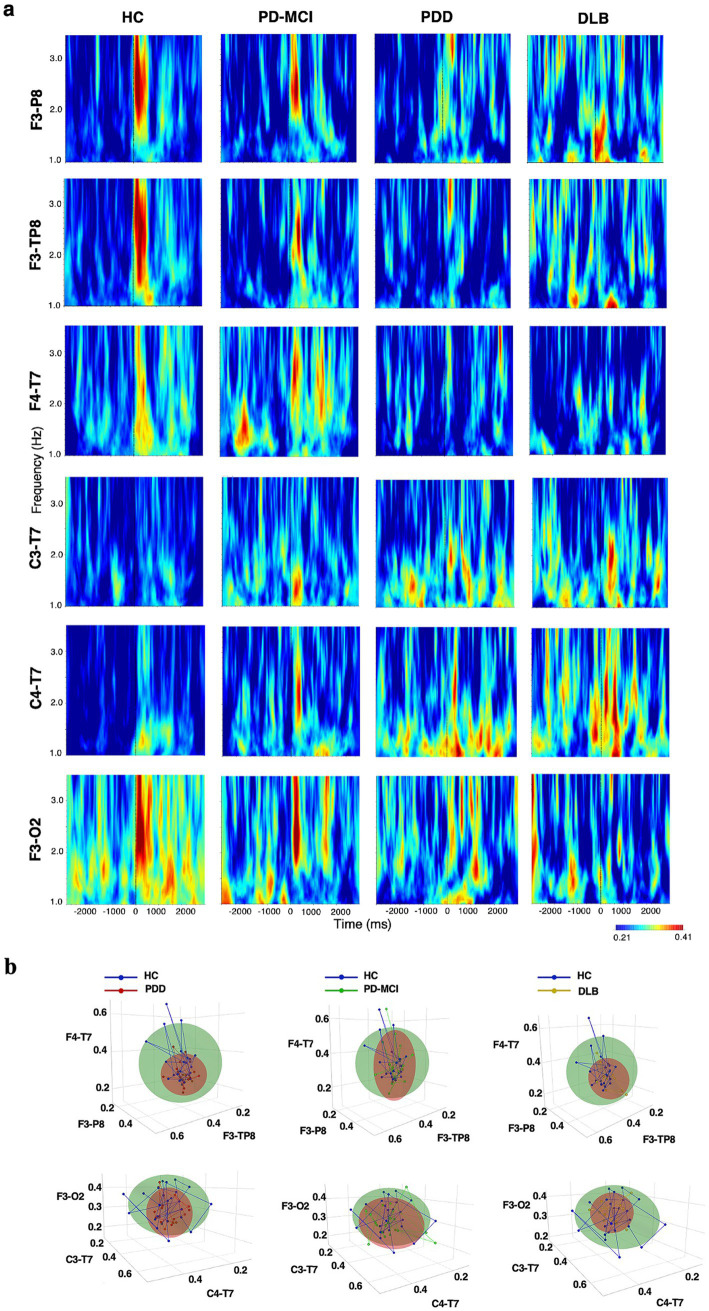
Delta band (1–3.5 Hz). **(a)** Wavelet spectrograms. Group-averaged wavelet time–frequency maps of event-related phase-locking value (PLV) across selected electrode pairs for each group (HC, PD-MCI, PDD, DLB). PLV values were first computed at the single-subject level and then averaged across subjects within each group. Warmer colors indicate stronger phase coherence. Time zero denotes stimulus onset. **(b)** Coherence feature embedding. 3D coherence embeddings illustrating group separation across the three most discriminative electrode pairs. Each point represents a single subject; ellipsoids denote within-group variability (±1 SD); connecting lines trace subject-specific trajectories across electrode-pair dimensions.

**Figure 3:**
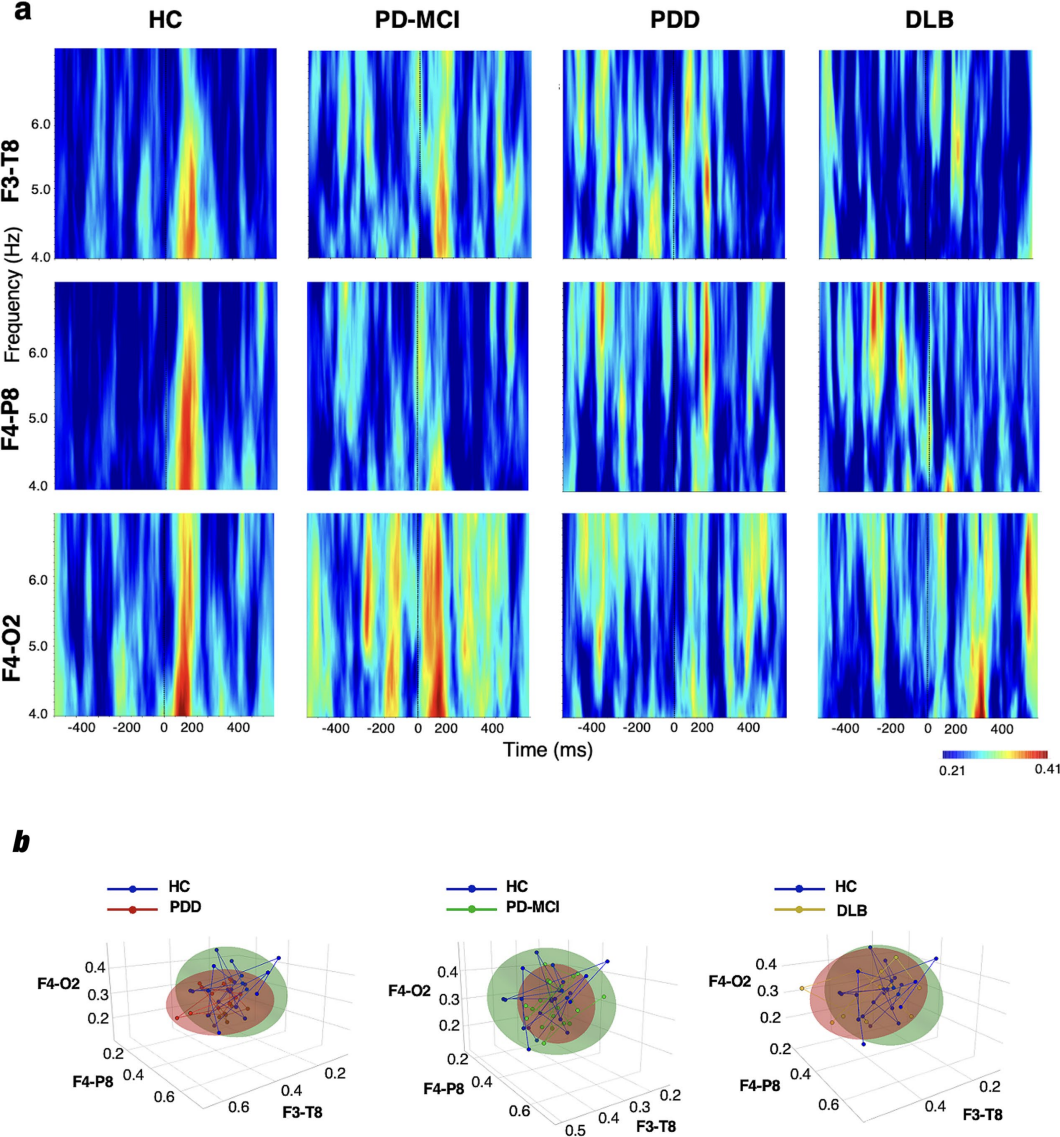
Theta band (4–7 Hz). **(a)** Wavelet spectrograms. Group-averaged wavelet time–frequency maps of event-related phase-locking value (PLV) across selected electrode pairs for each group (HC, PD-MCI, PDD, DLB). PLV estimates were computed per subject and then averaged across subjects within each group. Warmer colors indicate stronger coherence. Time zero denotes stimulus onset. **(b)** Coherence feature embedding. 3D coherence embeddings illustrating group separation across the three most discriminative electrode pairs. Each point represents a single subject; ellipsoids denote within-group variability (±1 SD); connecting lines trace subject-specific trajectories across electrode-pair dimensions.

In the following, we report group-level PLV results from a mixed-design repeated-measures ANOVA assessing event-related EEG phase coherence across groups and hemispheric locations. Wavelet spectrograms were subsequently used to visualize the temporal and spectral distribution of coherence across conditions (Lachaux et al., 2002; Keizer et al., 2010) (Figures 2a, 3a).

Supplementary Table S1 summarizes edge-level sensitivity analyses using bootstrapping and permutation testing, reporting effect sizes, confidence intervals, and permutation-derived *p*-values for all predefined electrode pairs for delta and theta bands.

#### 3.1.1 Delta band

The omnibus ANOVA results did not reveal a significant main effect of the group on delta phase coherence values [*F*(3, 69) = 2.283, *p* = 0.087, η^2^p = 0.090]. However, planned exploratory pairwise contrasts based on estimated marginal means (EMMs) revealed differences between the HC and PD-MCI groups (*p* = 0.048, *g* = 0.26), the HC and PDD groups (*p* = 0.035, *g* = 0.73), and the HC and DLB groups (*p* = 0.049, *g* = 0.54).

#### 3.1.2 Theta band

The omnibus ANOVA results did not show a significant main effect of the group on theta band coherence values [*F*(3, 69) = 1.188, *p* = 0.321, η^2^*p* = 0.040]. Nevertheless, EMM-based planned contrasts indicated exploratory differences between the HC and PDD groups (*p* = 0.035, *g* = 0.43), and the HC and DLB groups (*p* = 0.049, *g* = 0.30), whereas, the HC vs. PD-MCI groups did not (*p* > 0.05, *g* = 0.22) (Table 2).

Supplementary Figures S1, S2 displays group-wise average coherence across left, right, and interhemispheric measures.

### 3.2 Behavioral and cognitive correlations with phase coherence from EEG activity

To assess whether the observed group-level differences in the mentioned EEG coherence had meaningful behavioral and clinical correlates, we performed linear regression analyses using MMSE scores and error rates as outcome measures. All regression analyses were performed with age included as a covariate. A complete summary of standardized regression coefficients, explained variance, and significance levels for both overall and group-wise coherence–behavior associations is provided in Supplementary Table S5.

#### 3.2.1 Delta band

##### 3.2.1.1 Error scores

**Figure 4:**
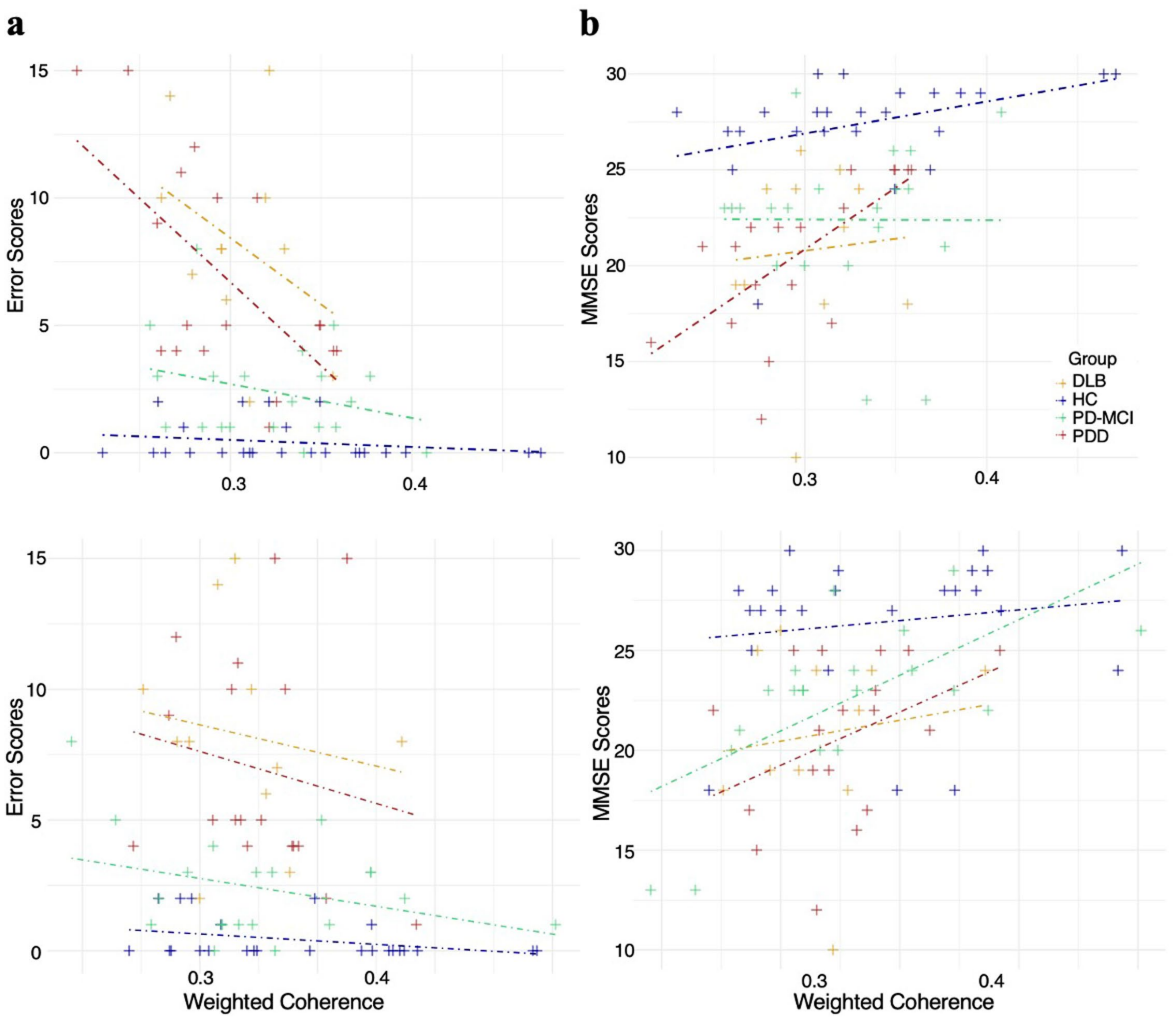
Linear regression of behavioral and cognitive performance with delta **(a)** and theta **(b)** band coherence. Scatter plots show associations between weighted average event-related coherence and behavioral error scores (top panels) or MMSE scores (bottom panels) for the delta **(a)** and theta **(b)** bands. Each marker represents a single participant. Weighted coherence values reflect participant-level averages computed across electrode pairs contributing to the regression model (see Methods).

In the delta band, both inter- and intrahemispheric event-related EEG coherence were significantly associated with error scores to the oddball target detection across participants. The overall regression model revealed a strong effect for intrahemispheric coherence (*R*^2^ = 0.610, β = −0.72, *p* < 0.001) and a moderate effect for interhemispheric coherence (*R*^2^ = 0.470, β = −0.61, *p* < 0.01). Group-wise analyses indicated that these associations were strongest in the PDD group (*R*^2^ = 0.670, β = −0.82, *p* < 0.001), followed by the PD-MCI (*R*^2^ = 0.330, β = −0.57, *p* < 0.05) and DLB (*R*^2^ = 0.298, β = −0.55, *p* < 0.05) groups (Figure 4a). Detailed results and confidence intervals for regression coefficients are provided in Table 2.

##### 3.2.1.2 MMSE

Delta-band event-related EEG coherence was significantly associated with MMSE scores across participants, for both interhemispheric (*R*^2^ = 0.300, β = 0.53, *p* < 0.05) and intrahemispheric electrode pairs (*R*^2^ = 0.335, β = 0.58, *p* < 0.05). Group-wise regressions showed that the association was strongest in the PDD group (*R*^2^ = 0.530, β = 0.73, *p* < 0.001), followed by PD-MCI (*R*^2^ = 0.330, β = 0.57, *p* < 0.05) and DLB (*R*^2^ = 0.155, β = 0.40, *p* > 0.05).

#### 3.2.2 Theta band

##### 3.2.2.1 Error scores

In the theta band, the overall regression model revealed a markedly significant association between intrahemispheric event-related EEG coherence and error scores (*R*^2^ = 0.460, β = −0.53, *p* < 0.05). In contrast, the interhemispheric model showed a mild but still significant effect (*R*^2^ = 0.350, β = −0.47, *p* < 0.05). Group-wise analyses indicated these relationships were most pronounced in the PDD group (*R*^2^ = 0.690, β = −0.83, *p* < 0.01), followed by the PD-MCI (*R*^2^ = 0.370, β = −0.61, *p* < 0.05) and DLB (*R*^2^ = 0.170, β = −0.41, *p* > 0.05) groups (Figure 4b).

##### 3.2.2.2 MMSE

Theta-band event-related EEG coherence showed a significant overall association with MMSE scores for both intrahemispheric (*R*^2^ = 0.388, β = 0.058, *p* < 0.01) and interhemispheric electrode pairs (*R*^2^ = 0.289, β = 0.077, *p* < 0.05). Within-group analyses revealed the strongest association in the PDD group (*R*^2^ = 0.430, *p* < 0.01 for intrahemispheric; *R*^2^ = 0.280, *p* < 0.05 for interhemispheric), followed by moderate effects in DLB (*R*^2^ = 0.230 and 0.290, respectively, both *p* < 0.05) (see Supplementary Table S2 for coherence values of the most discriminative electrode pairs, stratified by MMSE and error scores).

### 3.3 Functional network characterization

To further characterize the disruptions in cortical network models estimated from event-related EEG coherence, we applied multivariate embedding and network efficiency estimation to the EEG coherence values with the following core results.

Group differences in network topology are visualized both at the edge level (average coherence graphs; Supplementary Figures S3a, S4a) and at the global network level using graph-theoretic efficiency metrics (Supplementary Figures S3b, S4b), providing complementary views of disease-related network disruption.

#### 3.3.1 Coherence feature embedding

A 3D coherence embedding index based on the top three discriminative electrode pairs revealed clear group-level separation, utilizing inputs from event-related EEG coherence in the delta and theta bands. The most pronounced separation from the HC group was observed in the PDD (β = 0.60 to 0.77, *p* < 0.05) and DLB (β = 0.49 to 0.57, *p* < 0.05) groups using the coherence values in the delta band (Figure 2b). In the theta band, although qualitative differences in coherence distributions were visible across groups, statistically significant multivariate separation was observed only for the HC vs. PDD comparison (β = 0.58–0.65, *p* < 0.05; Figure 3b). As a methodological remark, the most discriminant electrode pairs were identified through preliminary signal analysis based on inter-group variance and retained for their discriminative strength in the multivariate coherence projection (Figure 3b).

#### 3.3.2 Network efficiency

Compared to the HC group, the global efficiency index estimated from EEG coherence in the delta band was lower in the PDD group (HC: 0.56, PDD: 0.38), reflecting a 31% reduction (*p* < 0.001). Furthermore, the PD-MCI group showed a 20% reduction (*p* < 0.05) and the DLB group a 27% reduction (*p* < 0.01). The local efficiency index from the delta coherence was also lower in PDD (−24%, *p* < 0.01) and DLB (−19%, *p* < 0.05) groups. In the theta band, the global efficiency index was lower by 27% in the PDD group and 22% in the DLB group (both *p* < 0.05) over the HC group. Finally, the small-worldness (*σ*) index was lower in the PDD group (σ = 1.04) compared to HC (σ = 1.92; *p* < 0.001). Group differences in network topology are visualized both at the edge level (average coherence graphs; Supplementary Figures S3a, S4a) and at the global network level using graph-theoretic efficiency metrics (Supplementary Figures S3b, S4b), providing complementary views of disease-related network disruption.

### 3.4 Supervised classification of dementia subtypes

LASSO logistic regression with 10-fold cross-validation was applied to classify event-related EEG delta coherence features between the current HC and clinical subgroups. In each fold, LASSO selected the most predictive coherence features (electrode pairs) at the optimal lambda, and model performance was evaluated using the area under the receiver operating characteristic (AUC) and confusion matrices derived from fold-wise predictions. Several of the top-ranked electrode pairs showing discriminant EEG delta coherence also appeared prominently in both time–frequency and network-level analyses, supporting the cross-method robustness of these features.

**Figure 5:**
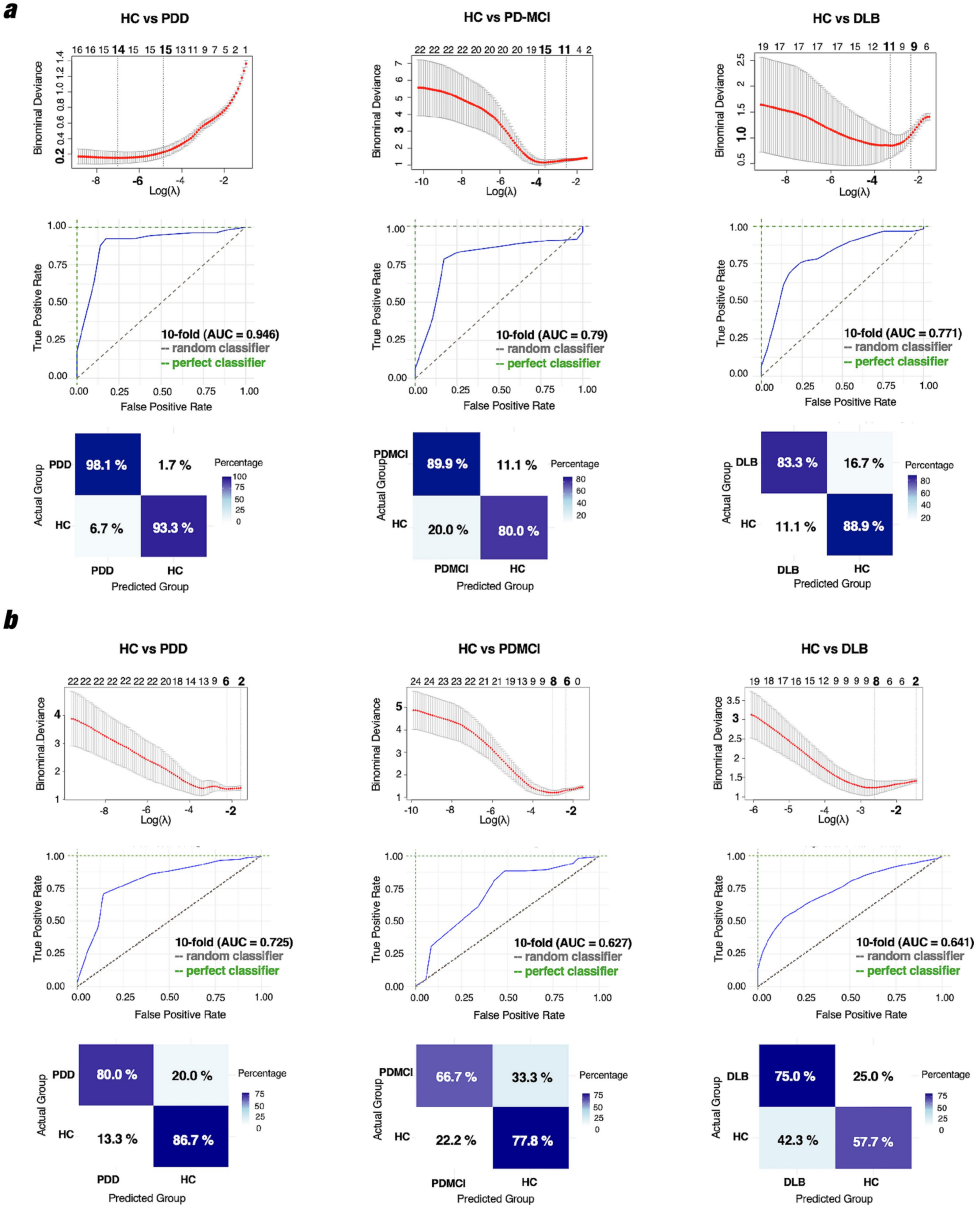
**(a)** Classification performance using delta **(a)** and theta **(b)** band EEG coherence features. LASSO was applied to distinguish HC from PD-MCI, PDD, and DLB using phase coherence features. Top panels show binomial deviance across log(λ) during 10-fold cross-validation. Middle panels display ROC curves with corresponding AUC values. Bottom panels show normalized confusion matrices indicating class-wise classification performance.

Across comparisons, delta-band coherence features consistently yielded more stable and multivariate classification solutions than theta-band coherence features, as reflected by higher values of K at λ_1se and stronger cross-validated performance metrics (Figure 5).

Confidence intervals for all AUC estimates were computed using bootstrap resampling and are reported in Supplementary Table S3.

#### 3.4.1 Delta band

##### 3.4.1.1 HC vs. PD-MCI

Using delta-band coherence features, the LASSO model retained K = 15 non-zero predictors at λ_min (at the optimal regularization parameter), while the more conservative λ_1se solution retained *K* = 11 predictors (Figure 5a), indicating a compact multivariate feature set supporting classification performance. For interpretability, the most influential predictors were identified based on the largest absolute regression coefficients. Negatively weighted features, such as F3–TP8 (β = −0.41, AUC = 0.70) and F4–T7 (β = −0.48, AUC = 0.79), reflected reduced fronto-parietal EEG coherence in the delta band in the PD-MCI group. Positively weighted pairs (e.g., C3–T7: β = 0.31, AUC = 0.58; C4–T7: β = 0.83, AUC = 0.63) indicated relatively preserved or enhanced EEG delta coherence in frontotemporal networks. At λ_min, the model achieved a cross-validated AUC of 0.790, with 89.9% sensitivity and 80.0% specificity.

Supplementary Table S1 summarizes sensitivity analyses using bootstrapping and permutation, confirming the robustness of cross-validated classification performance for delta and theta bands.

##### 3.4.1.2 HC vs. PDD

In the HC vs. PDD comparison, delta-band coherence features yielded a stable multivariate solution, with *K* = 15 predictors retained at λ_min and *K* = 14 predictors retained at λ_1se. LASSO identified the following six key electrode pairs with the most negatively weighted features from the EEG delta coherence: F3–TP8 (β = −0.97, AUC = 0.98), F3–P8 (β = −0.93, AUC = 0.92), F3-O2 (β = −0.82, AUC = 0.86), and F4–T7 (β = −0.69, AUC = 0.82). Positively weighted features from the EEG delta coherence were observed for the following electrode pairs: C3–T7 (β = 0.73, AUC = 0.88) and C4–T7 (β = 0.68, AUC = 0.75). The model achieved a classification performance with an AUC of 0.946, sensitivity of 98.1%, and specificity of 93.3% (Tables 3, 4).

**Table 3:** Classification results for delta band.

Comparison	F1 score	Precision	Sensitivity	Specificity	Min lambda: 1-SE lambda	AUC overall	LASSO (coefficient)	AUC (mean ± SD)
HC–PD-MCI	86.4%	83.2%	89.9%	80.0%	0.0214: 0.0787	79.0%	F4-T7 (−0.48)	0.79 ± 0.26
							C3-T7 (0.31)	0.78 ± 0.32
							F3-P8 (−0.19)	0.74 ± 0.21
							F3-O2 (−0.18)	0.73 ± 0.24
							C4-T7 (0.83)	0.72 ± 0.28
							F3-TP8 (−0.41)	0.70 ± 0.17
HC–PDD	96.9%	95.4%	98.1%	93.3%	0.0028: 0.0161%	94.6%	F3-TP8 (−0.97)	0.98 ± 0.10
							F3-P8 (−0.93)	0.92 ± 0.11
							C3-T7 (0.73)	0.88 ± 0.15
							F3-O2 (−0.82)	0.86 ± 0.16
							F4-T7 (−0.69)	0.82 ± 0.14
							C4-T7 (0.68)	0.75 ± 0.17
HC–DLB	84.6%	80.1%	83.3%	88.9%	0.0446: 0.1029%	77.1%	C3-T7 (0.90)	0.88 ± 0.21
							F3-O2 (−0.58)	0.80 ± 0.15
							C4-T7 (0.84)	0.79 ± 0.19
							F4-T7 (−0.61)	0.77 ± 0.21
							F3-TP8 (−0.43)	0.74 ± 0.18
							F3-P8 (−0.35)	0.59 ± 0.21

**Table 4:** Classification results for theta band.

Comparison	F1 score	Precision	Sensitivity	Specificity	Min lambda: 1-SE lambda	AUC overall	LASSO (coefficient)	AUC (mean ± SD)
HC–PD-MCI	77.8%	66.7%	77.8%	77.8%	0.0490: 0.0940%	62.7%	F4-P8 (−0.86)	0.87 ± 0.16
							F3-T8 (−0.70)	0.81 ± 0.19
							F4-O2 (−0.31)	0.67 ± 0.34
HC–PDD	86.4%	83.2%	89.9%	80.0%	0.1052: 0.2019%	72.5%	F4-P8 (−0.90)	0.83 ± 0.20
							F4-O2 (−0.72)	0.71 ± 0.17
							F3-T8 (−0.53)	0.70 ± 0.29
HC–DLB	57.7%	75.0%	57.7%	42.3%	0.0732: 0.2342%	64.1%	F4-P8 (−0.86)	0.89 ± 0.24
							F3-T8 (−0.74)	0.85 ± 0.26
							F4-O2 (−0.46)	0.67 ± 0.34

Features selected by LASSO (AUC ≥ 0.75) in the HC vs PDD comparison were identified via 10-fold cross-validation, ranked by AUC, and examined across other group contrasts. For the theta band, three top-ranked features were reported similarly.

##### 3.4.1.3 HC vs. DLB

For the HC vs. DLB comparison in the delta band, the LASSO model retained K = 10 predictors at λ_min and K = 9 predictors at λ_1se. Negatively weighted features from the EEG delta coherence were observed for the following electrode pairs: F3-TP8 (β = −0.43, AUC = 0.74), F3-P8 (β = −0.35, AUC = 0.59), F3–O2 (β = −0.58, AUC = 0.80), and F4-T7 (β = −0.61, AUC = 0.77), while C3–T7 (β = 0.90, AUC = 0.88) and C4–T7 (β = 0.84, AUC = 0.79) emerged as the strongest positively contributing pairs. At λ_min, the model achieved a cross-validated AUC of 0.771, with 83.3% sensitivity and 88.9% specificity.

#### 3.4.2 Theta band

##### 3.4.2.1 HC vs. PD-MCI

In the theta band, the LASSO model retained *K* = 8 predictors at λ_min, while the λ_1se solution retained *K* = 6 predictors (Figure 5b), indicating a compact but less stable multivariate feature set relative to delta-band models. Negatively weighted features from the EEG theta coherence were observed for the following electrode pairs: F4-P8 (β = −0.86, AUC = 0.87) and F3–T8 (β = −0.70, AUC = 0.81). At λ_min, the model achieved a cross-validated AUC of 0.627, with 66.7% sensitivity and 77.8% specificity.

##### 3.4.2.2 HC vs. PDD

Theta-band coherence provided a sparse and unstable discriminative signal for the HC vs. PDD comparison. At λ_min, the model retained K = 4 predictors, whereas the λ_1se solution was intercept-only (*K* = 0). LASSO identified three key electrode pairs for the EEG theta coherence, with the strongest negative beta weights observed in F4–P8 (β = −0.90, AUC = 0.83), F4–O2 (β = −0.72, AUC = 0.71), and F3–T8 (β = −0.53, AUC = 0.70). Despite sparsity under λ_1se, the λ_min model achieved a cross-validated AUC of 0.725, with 89.9% sensitivity and 80.0% specificity. As detailed in Supplementary Table S3, several fronto-temporal connections (e.g., F3–TP8, F4–T7) also showed high univariate discriminative ability, with AUC values exceeding 0.9.

##### 3.4.2.3 HC vs. DLB

Similarly, theta-band classification of HC vs. DLB yielded a sparse and unstable solution. The model retained *K* = 8 predictors at λ_min, whereas the λ_1se solution was intercept-only (*K* = 0). Key features, with negatively weighted pairs for the EEG theta coherence were F4–P8 (β = −0.86, AUC = 0.89), F4–O2 (β = −0.46, AUC = 0.67), and F3–T8 (β = −0.74, AUC = 0.85). At λ_min, the model achieved a cross-validated AUC of 0.641, with 75.0% sensitivity and 57.7% specificity.

### 3.5 Sensitivity analyses and validation

To assess classification robustness, we conducted 5,000-iteration bootstrapping and permutation testing on the above event-related EEG coherence features.

#### 3.5.1 Delta band

Compared to the HC group, the PDD group showed lower event-related EEG coherence in the delta band at the frontotemporal electrode pairs, including F3–TP8 (*p* = 0.016, AUC = 0.98 ± 0.10), F3–P8 (*p* = 0.012, AUC = 0.92 ± 0.11), F3–O2 (*p* = 0.032, AUC = 0.86 ± 0.16), and F4–T7 (*p* = 0.006, AUC = 0.82 ± 0.14). Significant reductions in EEG delta coherence features were also observed in the PD-MCI (F4–T7; *p* = 0.032, AUC = 0.79 ± 0.26) and DLB (F3-TP8; *p* = 0.045, AUC = 0.74 ± 0.18) groups.

#### 3.5.2 Theta band

Compared to the HC group, the PDD group also showed lower event-related EEG coherence in the delta band, especially at the right frontotemporal electrode pairs, including F4–P8 (*p* = 0.012, AUC = 0.83 ± 0.20) and F4–O2 (*p* = 0.011, AUC = 0.71 ± 0.17). Significant reductions in EEG theta coherence features were also observed in the PD-MCI (F4–P8, *p* = 0.046, AUC = 0.47 ± 0.33; F3–T8, *p* = 0.048, AUC = 0.81 ± 0.19) and DLB (F4–P8, *p* = 0.049, AUC = 0.89 ± 0.24; F4–O2, *p* = 0.045, AUC = 0.67 ± 0.34) groups. Full electrode-level results are presented in Supplementary Table S1 and Supplementary Figures S1, S2.

## 4 Discussion

This study evaluated the utility of delta- and theta-band event-related oscillatory (ERO) phase coherence between scalp electrode pairs for differentiating HC, PD-MCI, PDD, and DLB participants during visual oddball detection using ML models. Analyses conducted at both the group and individual (classification) levels showed that delta-band ERO coherence was particularly sensitive to cognitive status, with significant associations observed with oddball-task performance and clinical severity measures.

These results align with and extend prior EEG-based ML studies reporting high discriminative power using brain functional connectivity–informed features (Yang et al., 2023; Pirrone et al., 2022). Together, they demonstrate frequency- and network-specific alterations in task-evoked EEG coherence across PD-MCI, PDD, and DLB.

From a clinical perspective, these findings suggest that task-evoked delta-band EEG coherence may capture disease-related network dysfunction that is not fully reflected by behavioral performance alone. Consistent with this interpretation, the ML models achieved high classification performance in distinguishing HC participants from patient groups (accuracy > 95.7%), motivating future studies to explore the feasibility of stratifying PD and DLB patients based on disruptions in cortical functional connectivity relevant to cognitive processes.

Disruption in delta-band ERO coherence across interhemispheric and intrahemispheric pathways was evident in the PDD group, with particularly pronounced reductions in right frontotemporal and frontoparietal electrode pairs, accompanied by significantly reduced global network efficiency and impaired local efficiency. These findings suggest a broad disruption of both long-range cortical integration and short-range communication.

In contrast, increased centro-temporal delta-band ERO coherence was observed in the PDD and DLB groups relative to the HC group. While such increases may reflect compensatory synchronization aimed at preserving residual cognitive–motor integration (Cabeza et al., 2002), they may alternatively indicate maladaptive hyperconnectivity arising from impaired inhibitory control or reduced thalamic gating (Fries, 2005; Uhlhaas and Singer, 2010). Consistent with this interpretation, aberrant delta-band EEG activity in temporo-parietal networks has been associated with inefficient recruitment of attentional and sensorimotor resources in early stages of dementia (Başar, 2013), a pattern that may be further amplified in PDD and DLB due to more widespread cortical cholinergic dysfunction (Ballinger et al., 2016).

Viewed at the network level, these connection-specific coherence alterations were accompanied by a consistent reduction in global efficiency across patient groups, most pronounced in PDD and DLB, indicating impaired large-scale functional integration. In contrast, reductions in local efficiency were less uniform and emerged selectively in more cognitively impaired phenotypes. This dissociation is consistent with established interpretations of EEG functional networks, whereby global efficiency reflects long-range integrative capacity, whereas local efficiency indexes neighborhood-level segregation. The predominance of global efficiency reductions provides a coherent framework for interpreting the coexistence of focal coherence decreases and regional increases.

Delta-band ERO coherence in PD and DLB patients was closely linked to behavioral and cognitive performance, showing negative correlations with oddball-task error rates and positive correlations with MMSE scores within the PDD group, with the strongest associations observed at frontotemporal and frontoparietal electrode pairs. This coupling between delta-band coherence and cognitive performance supports the interpretation of low-frequency network synchronization as a mechanistically relevant marker of attentional and executive system integrity.

The phase-locking value (PLV) of EEG (or MEG) oscillatory activity provides a nonlinear, time-resolved measure of pairwise connectivity (Lachaux et al., 1999; Handayani et al., 2018) by capturing phase consistency between signals generated by distributed neural assemblies, thereby indexing functional coupling among cortical regions underlying cognitive processing. Despite growing recognition of PLV as a promising tool for characterizing functional integration in the aging brain, its use in Parkinson’s disease cohorts has been comparatively underexplored (Mormann et al., 2000; Blinowska et al., 2013).

Related work has begun to demonstrate the utility of EEG-based connectivity measures in neurodegenerative conditions, providing important groundwork. For example, Fide et al. (2023) applied ERO imaginary coherence (ICoh) during oddball task-related and resting-state conditions to investigate cortical functional disconnection in Alzheimer’s disease and amnestic mild cognitive impairment. Similarly, Yıldırım et al. (2024) reported reduced delta- and theta-band ERO responses during oddball tasks in PDD and DLB, consistent with frontal executive dysfunction.

Consistent with prior literature, the present results indicate that task-evoked delta (1–3.5 Hz) and theta (4–7 Hz) phase synchrony during oddball paradigms reflects large-scale neural communication supporting target detection, attention allocation, decision making, and executive control rather than purely motor processes. Alterations in these low-frequency dynamics in Parkinsonian and Lewy-body spectrum dementias are therefore interpreted as markers of network-level disconnection associated with disease-related cognitive phenotypes (Schulman et al., 2011).

Although alpha and beta frequency bands have also been implicated in cognitive processing, beta-band activity in Parkinson’s disease is most commonly examined in relation to motor circuitry and dopaminergic treatment effect (Mathiopoulou et al., 2024; Melgari et al., 2014; Martín-Rodríguez et al., 2025), given its strong modulation by dopaminergic state, which complicates interpretation in task-based cognitive paradigms. Alpha-band alterations, while linked to cognitive decline (Zhao et al., 2025; Benz et al., 2014), are more frequently characterized using spectral power or resting-state measures rather than event-related, phase-based connectivity metrics. Accordingly, the present study emphasized delta and theta phase synchrony as robust indices of stimulus-locked, large-scale network coordination during oddball target detection.

Within this framework, the ERO coherence features that most clearly differentiated PD and DLB patients from healthy controls were concentrated in the delta band, particularly within fronto-parietal and fronto-temporal electrode pairs. Delta-band responses during oddball tasks are closely associated with large-scale cortical networks supporting attentional and executive control and may be tightly linked to thalamocortical loop integrity (Başar et al., 1999; Harmony, 2013). The reduced delta coherence observed in the present PDD and DLB cohorts converges with prior fMRI evidence demonstrating pronounced within-network connectivity loss in frontal, temporal, and motor systems, with relative sparing of between-network and default-mode interactions (Schumacher et al., 2018), as well as diminished fronto-thalamic connectivity in α-synucleinopathies. Together, these findings position delta-band ERO coherence as a sensitive marker of task-evoked network dysfunction in Parkinsonian and Lewy-body spectrum dementias.

Beyond frequency- and connection-specific effects, network-level analyses revealed both shared and disease-specific patterns of functional network disruption. Parkinson’s disease–related dementia (PDD) was characterized by marked reductions in global efficiency and small-worldness in the delta and theta bands, indicating a widespread loss of functional integration consistent with progressive disconnection of large-scale cortico–cortical and cortico–subcortical networks. In contrast, dementia with Lewy bodies (DLB) exhibited pronounced but less uniform reductions in global efficiency, accompanied by relatively preserved small-world characteristics compared with PDD, suggesting a network organization in which global integration is compromised while local clustering remains partially maintained.

At an interpretive level, the concurrent reductions in local efficiency and the emergence of spatially specific discriminative electrode pairs indicate that global network disintegration is accompanied by regionally accentuated vulnerabilities. This pattern likely reflects heterogeneous propagation of α-synuclein pathology and neuromodulatory deficits across cortical networks. Collectively, these findings support a conceptual model in which PDD is characterized by a more homogeneous and severe global network collapse, whereas DLB presents a mixed pattern of global impairment with comparatively preserved local network organization.

The following mechanistic interpretations are necessarily inferential and are intended to provide a theoretical framework linking the observed EEG coherence alterations to known thalamocortical and neurotransmitter-level mechanisms. Beyond the empirical findings, and at a theoretical level, consistent with established EEG and MEG literature, it can be speculated that the disrupted delta-band ERO coherence observed in PDD, PD-MCI, and DLB reflects a form of thalamocortical dysrhythmia associated with impaired cortico–cortical and cortico–subcortical communication (Başar and Güntekin, 2008). While thalamocortical dysrhythmia cannot be directly measured using scalp EEG, alterations in low-frequency delta and theta phase synchronization have been widely interpreted as noninvasive markers of disrupted thalamocortical communication and impaired large-scale network coordination.

Within this framework, event-related delta oscillations are known to index attentional and cognitive control loops, and their attenuation may reflect reduced temporal synchronization across distributed cortical populations engaged during target detection. The thalamocortical dysrhythmia model proposed by Llinás et al. (2005) attributes such low-frequency desynchronization to impaired GABAergic modulation within thalamic reticular circuits, leading to destabilized thalamocortical rhythmic generators and diminished coordination of cortical cognitive networks.

Converging neurophysiological evidence further supports this interpretation. Transcranial magnetic stimulation studies across the Parkinson’s disease dementia and Alzheimer’s disease continuum demonstrate reductions in intracortical inhibition and altered facilitation, indicating a shift toward cortical hyperexcitability driven by weakened GABAergic and cholinergic tone (Cantello et al., 2002; Lefaucheur, 2005; Ni and Chen, 2015; Leon-Sarmiento et al., 2013). Pharmacological TMS experiments show that dopaminergic and GABAergic agents can transiently normalize inhibitory deficits (Pierantozzi et al., 2001), underscoring the role of neurotransmitter dysfunction in shaping cortical network dynamics (Ziemann et al., 2015). Collectively, these findings link neurotransmitter-level failure—particularly involving GABA, acetylcholine, and dopamine—to impaired thalamocortical gating and fronto-temporal network instability, providing a plausible cellular and circuit-level substrate for the delta-band coherence breakdown observed in the present EEG data.

### 4.1 Methodological remarks

This exploratory study was conducted under several methodological constraints, the most notable being the sample size. The overall cohort consisted of 73 participants (24 HC, 20 PD-MCI, 18 PDD, 11 DLB), with subgroup comparisons limited by modest group sizes, particularly in the DLB arm. These numbers reflect the practical challenges of recruiting well-characterized neurodegenerative samples (van Dellen et al., 2015) under tightly controlled clinical and electrophysiological conditions. Importantly, inclusion and exclusion criteria were deliberately stringent to reduce clinical heterogeneity and maximize internal validity as a reference for future studies. All participants underwent structured diagnostic evaluation and task-based EEG recording rather than resting-state acquisition, which further constrained enrollment. In addition, rigorous quality-control procedures—including thresholds for EEG signal-to-noise ratio, electrode impedance, and artifact rejection—led to the exclusion of unusable recordings, prioritizing data reliability over quantity.

In addition to general recruitment constraints, subgroup-specific considerations were applied to the DLB cohort. Visual hallucinations are a core clinical feature of dementia with Lewy bodies, as defined by the Fourth Consensus Report of the DLB Consortium (McKeith et al., 2017), which identifies “recurrent visual hallucinations that are typically well formed and detailed” as a diagnostic core feature. Such hallucinations occur in up to ~80% of DLB patients and often emerge early in the disease course. Accordingly, to ensure symptom-level homogeneity within the DLB group and to enhance alignment between core clinical features and the visual nature of the oddball task, visual hallucinations were treated as a core inclusion criterion. Hallucination severity was assessed using the University of Miami Parkinson’s Disease Hallucinations Questionnaire (UM-PDHQ) (Andersson et al., 2008), and only patients with confirmed visual hallucinations (UM-PDHQ scores between 2 and 9) were included. Patients with absent, negligible, or non-visual hallucinations were excluded to minimize subgroup heterogeneity and potential bias in group-level comparisons (Agbomi et al., 2022).

Sex imbalance was also present in the sample, particularly in the PDD group (17 men, 1 woman). This imbalance reflects known sex distributions in PDD and DLB populations (Daniel and Lees, 1993), but nonetheless limits the generalizability of the present findings. In contrast, other key demographic variables, including age and education level, did not differ significantly between groups (*p* > 0.05), reducing the likelihood that these factors confounded group-level effects. Furthermore, all PD and DLB patients were maintained on stable dopaminergic medication regimens, ensuring pharmacological consistency across clinical groups (Emre et al., 2007). Where applicable, age-adjusted analyses were conducted for regression-based associations between EEG coherence and behavioral or cognitive measures, and these adjustments did not alter the direction or significance of the reported effects. The current dataset represents a tightly standardized and diagnostically rigorous cohort, suitable for the exploratory aims of this study. The study results provide a foundation for several future research directions.

Because supervised classification performance can be influenced by unequal group sizes, class imbalance represents a potential source of bias in the present analyses. To mitigate this effect, classification performance was primarily summarized using the threshold-independent area under the ROC curve (AUC), with class-specific sensitivity and specificity reported for each binary comparison. Feature selection was performed within each cross-validation fold using LASSO regularization to limit model complexity and reduce overfitting. Nevertheless, the modest and unequal subgroup sizes may still contribute to variability in fold-wise estimates. Future studies using larger, more balanced cohorts and imbalance-aware evaluation strategies will therefore be necessary to confirm generalizability (Varoquaux, 2018). Expanding sample size would also enable more granular modeling of coherence patterns within specific subtypes and symptom clusters, such as hallucination severity or executive dysfunction. Despite these limitations, the current dataset represents a strictly standardized and diagnostically rigorous cohort, well suited to the exploratory aims of this study and providing a solid foundation for future research.

The absence of a significant omnibus ANOVA effect alongside significant planned post-hoc comparisons likely reflects heterogeneity in group differences rather than an inconsistency in the results. Omnibus F-tests evaluate global between-group variance and may have reduced sensitivity when effects are concentrated in specific comparisons (e.g., HC vs. PDD and HC vs. DLB) but are smaller or absent in others (e.g., HC vs. PD-MCI) (Haans, 2018; Maxwell and Delaney, 2004). In such cases, *a priori* planned contrasts provide a more focused and statistically appropriate test of hypothesis-driven group differences (Schad et al., 2018; Keppel and Wickens, 2004).

A further limitation concerns the cross-sectional design. Although inherently cross-sectional, the inclusion of both PD-MCI and PDD groups—each analyzed in relation to healthy controls—permits a preliminary, stage-informed perspective on coherence alterations across the Parkinsonian cognitive continuum. However, future studies should aim to replicate these connectivity–performance associations in independent samples, ideally using longitudinal designs to determine whether such coherence disruptions track disease progression and cognitive decline over time.

In this context, the ERO coherence features identified through regularized classification and regression (e.g., interhemispheric frontotemporal pairs) may represent candidate markers for stratifying PD and DLB patients according to the degree of impairment in neurophysiological oscillatory mechanisms underlying cognitive information processing or subtype differentiation, pending external replication. Task-based EEG offers a scalable, non-invasive, and time-efficient approach for capturing functional network disruptions. Integration of ERO coherence metrics with other neurophysiological or imaging modalities at higher spatial resolution—such as MEG or functional magnetic resonance imaging—could further test and refine the present findings for clinical application. With appropriate validation, such multimodal approaches may contribute to future frameworks for individualized monitoring, clinical decision support, or stratified intervention in neurodegenerative disorders.

Finally, future work should consider the role of pharmacological and non-pharmacological modulation of ERO coherence metrics to further probe underlying neurophysiological mechanisms. In the present study, all patients were recorded during their regular dopaminergic medication cycles, although timing relative to drug intake was not tightly controlled. While dopaminergic medication reliably modulates beta-band activity in Parkinson’s disease, low-frequency event-related delta/theta markers linked to cognitive processing often show heterogeneous ON/OFF effects and are commonly interpreted as reflecting network-level cognitive dysfunction that does not necessarily normalize with dopaminergic therapy (Tinkhauser et al., 2017; Sharma et al., 2021; Kiani et al., 2023). Nevertheless, medication effects cannot be fully excluded and should be considered a limitation of the present study. Although this reflects real-world clinical variability, future studies could systematically compare on- and off-medication states to clarify the influence of dopaminergic treatment on phase coherence, particularly in cognitively impaired subgroups88.

## 5 Conclusion

In the present study, machine-learning models using oddball-related ERO delta-phase coherence between fronto-posterior electrode pairs achieved an overall classification accuracy exceeding 95% for differentiating patients with PDD from HC participants, and approximately 85% for differentiating PD-MCI and DLB from controls. Furthermore, abnormalities in ERO delta coherence were significantly associated with oddball task performance and global cognitive status in PD and DLB patients. Together, these findings highlight the potential of non-invasive EEG-based ERO delta PLV, combined with standard ML models, as an adjunct to standard clinical assessment for capturing and accurately classifying large-scale network dysfunction across the α-synucleinopathy spectrum, with translational relevance for clinical workup and outpatient screening, monitoring disease progression, and evaluating treatment-related changes not captured by behavioral performance or global cognitive scores in PD and DLB.

## Data Availability

The data analyzed in this study is subject to the following licenses/restrictions: the datasets analyzed during the current study are not publicly available due to participant confidentiality and institutional regulations but are available from the corresponding author on reasonable request and subject to ethical approval. Requests to access these datasets should be directed to Bahar Güntekin, bguntekin@medipol.edu.tr.
