# Supplementary material for: Wavelet event-related EEG phase coherence as a discriminant biomarker of the cognitive status in Parkinson’s and Lewy body disease
Source: Front Hum Neurosci. 2026 Apr 2;20:1696861. doi: 10.3389/fnhum.2026.1696861 (PMC13083073; doi:10.3389/fnhum.2026.1696861)
Supplement: Supplementary file 1 [file Table_1.pdf]

Supplementary Table 1.1. DELTA - HC vs PDMCI Bootstrapping and Permutation Results (Sensitivity Analysis and Validation)

**HC vs PDMCI**	**Mean Difference (Lower CI : Upper CI)**	**Effect Size (Lower Effect CI : Upper Effect CI)**	**Observed Difference (p-value)**
C3-T7	-0.054 (-0.106 : -0.004)	-0.695 (-0.106 : -0.004)	-0.053 **(p = 0.032*)**
F4-TP8	0.077 (0.017 : 0.144)	0.687 (0.017 : 0.144)	0.077 **(p = 0.029*)**
F4-P8	0.068 (0.008 : 0.128)	0.633 (0.008 : 0.128)	0.070 **(p = 0.048*)**
F3-O2	0.038 (-0.007 : 0.082)	0.548 (-0.007 : 0.82)	0.037 (p = 0.099)
F3-TP8	0.055 (-0.002 : 0.112)	0.538 (-0.002 : 0.112)	0.055 (p = 0.094)
C3-O2	-0.054 (-0.101 : -0.004)	-0.537 (-0.101 : 0.008)	-0.048 (p = 0.088)
F4-TP7	-0.047 (-0.012 : 0.008)	0.491 (-0.012 : 0.128)	0.056 (p = 0124)
F3-P8	0.055 (-0.009 : 0.095)	0.479 (-0.009 : 0.095)	0.043 (p = 0.132)
C4-T8	0.044 (-0.021 : 0.086)	0.423 (-0.021 : 0.086)	0.034 (p = 0.235)
C4-TP8	0.036 (-0.018 : 0.085)	0.412 (-0.018 : 0.085)	0.031 (p = 0.220)
F4-P4	0.034 (-0.017 : 0.086)	0.401 (-0.017 : 0.086)	0.034 (p = 0.212)
F4-T7	0.030 (-0.043 : 0.100)	0.274 (-0.043 : 0.100)	0.031 (p = 0.379)
C3-T8	-0.033 (-0.085 : 0.018)	-0.389 (-0.085 : 0.018)	-0.032 (p = 0.220)
F3-P7	0.044 (-0.021 : 0.116)	0.376 (-0.021 : 0.116)	0.046 (p = 0.208)
C4-P4	0.033 (-0.026 : 0.087)	0.336 (-0.026 : 0.087)	0.032 (p = 0.296)
C3-P8	-0.027 (-0.079 : 0.023)	-0.298 (-0.079 : 0.023)	-0.026 (p = 0.333)
F4-P3	0.032 (-0.031 : 0.095)	0.289 (-0.031 : 0.095)	0.031 (p = 0.362)
C3-TP8	-0.033 (-0.087 : 0.024)	-0.390 (-0.087 : 0.024)	-0.033 **(p = 0.032*)**
F4-O2	0.023 (-0.032 : 0.083)	0.262 (-0.032 : 0.083)	0.021 (p = 0.452)
C4-P7	0.026 (-0.039 : 0.088)	0.256 (-0.039 : 0.088)	0.027 (p = 0.792)
F4-P7	0.033 (-0.037 : 0.104)	0.244 (-0.037 : 0.104)	0.032 (p = 0.417)
C3-TP7	-0.025 (-0.091 : 0.032)	-0.240 (-0.091 : 0.032)	-0.027 (p = 0.365)
F3-T7	-0.023 (-0.086 : 0.038)	-0.230 (-0.086 : 0.038)	-0.022 (p = 0.032)
F4-O1	-0.024 (-0.049 : 0.097)	0.219 (-0.049 : 0.097)	0.022 (p = 0.469)
F3-O1	0.017 (-0.067 : 0.039)	-0.214 (-0.067 : 0.039)	-0.017 (p = 0.537)
C4-P3	0.024 (-0.046 : 0.087)	0.211 (-0.046 : 0.087)	0.025 (p = 0.497)
C4-TP7	0.018 (-0.035 : 0.073)	0.202 (-0.035 : 0.073)	0.017 (p = 0.524)
C4-P8	0.014 (-0.039 : 0.064)	0.166 (-0.038 : 0.064)	0.014 (p = 0.600)
F4-T8	0.015 (-0.048 : 0.075)	0.148 (-0.048 : 0.073)	0.015 (p = 0.621)
C3-P3	0.013 (-0.050 : 0.073)	0.132 (-0.050 : 0.074)	0.012 (p = 0.693)
F3-TP7	0.013 (-0.042 : 0.076)	0.124 (-0.042 : 0.076)	0.011 (p = 0.712)
F3-P4	0.008 (-0.041 : 0.056)	0.097 (-0.041 : 0.056)	0.008 (p = 0.739)
C3-P7	0.008 (-0.051 : 0.066)	0.089 (-0.051 : 0.066)	0.007 (p = 0.792)
C3-O1	-0.009 (-0.072 : 0.055)	-0.079 (-0.072 : 0.055)	-0.009 (p = 0.780)
C4-O2	-0.004 (-0.059 : 0.056)	-0.047 (-0.059 : 0.056)	-0.002 (p = 0.915)
C4-T7	0.003 (-0.046 : 0.052)	0.044 (-0.046 : 0.052)	0.003 **(p = 0.032*)**
F3-T8	0.004 (-0.059 : 0.064)	0.044 (-0.059 : 0.064)	0.002 (p = 0.935)
C4-O1	0.000 (-0.061 : 0.064)	-0.013 (-0.1061 : 0.064)	0.000 (p = 0.999)
C3-P4	-0.001 (-0.050 : 0.043)	-0.013 (-0.050 : 0.043)	-0.002 (p = 0.924)
F3-P3	0.000 (-0.071 : 0.071)	-0.006 (-0.071 : 0.071)	0.001 (p = 0.973)

* Sensitivity values were computed across 10-fold cross-validation. Mean differences and 95 % confidence intervals were estimated using 5,000 bootstrap iterations. Observed group differences and corresponding p-values were derived from permutation testing (5,000 iterations, FDR-corrected). Effect sizes reflect Cohen's d, based on observed group-level differences.

(continue) DELTA - HC vs PDD Bootstrapping and Permutation Results (Sensitivity Analysis and Validation)

HC vs PDD	Mean Difference (Lower CI : Upper CI)	Effect Size (Lower Effect CI : Upper Effect CI)	Observed Difference (p-value)
F4-P8	0.120 (0.053 : 0.184)	1.089 (0.053 : 0.184)	0.119 **(p = 0.002∗)**
F4-TP8	0.121 (0.054 : 0.184)	1.067 (0.054 : 0.184)	0.119 **(p = 0.002∗)**
F4-T7	0.084 (0.028 : 0.142)	0.896 (0.028 : 0.142)	0.084 **(p = 0.006∗)**
C4-TP8	0.074 (0.020 : 0.128)	0.876 (0.020 : 0.128)	-0.005 **(p = 0.011∗)**
F3-P8	0.081 (0.020 : 0.137)	0.868 (0.020 : 0.137)	0.080 **(p = 0.012∗)**
F3-TP8	0.084 (0.021 : 0.144)	0.825 (0.021 : 0.144)	0.083 **(p = 0.016∗)**
F4-P4	0.066 (0.013 : 0.117)	0.780 (0.013 : 0.117)	0.065 **(p = 0.017∗)**
F3-P7	0.085 (0.025 : 0.149)	0.766 (0.025 : 0.149)	0.085 **(p = 0.017∗)**
C4-P3	0.075 (0.013 : 0.137)	0.720 (0.013 : 0.137)	0.076 **(p = 0.030∗)**
F3-O2	0.049 (0.005 : 0.092)	0.703 (0.005 : 0.092)	0.048 **(p = 0.035∗)**
C4-P8	0.054 (0.005 : 0.105)	0.690 (0.005 : 0.105)	0.053 **(p = 0.038∗)**
C4-P7	0.067 (0.013 : 0.124)	0.680 (0.013 : 0.124)	0.065 **(p = 0.047∗)**
C4-TP7	0.051 (0.004 : 0.100)	0.647 (0.004 : 0.100)	0.051 **(p = 0.042∗)**
F4-P7	0.076 (-0.001 : 0.149)	0.563 (-0.001 : 0.149)	0.075 **(p = 0.017∗)**
C4-O1	0.046 (-0.003 : 0.100)	0.518 (-0.003 : 0.100)	0.047 (p = 0.100)
C3-T7	-0.033 (-0.075 : 0.010)	-0.515 (-0.075 : 0.010)	-0.032 (p = 0.616)
F4-TP7	0.058 (-0.004 : 0.121)	0.506 (-0.004 : 0.121)	0.075 (p = 0.116)
F4-O1	0.048 (-0.007 : 0.103)	-0.695 (-0.007 : 0.103)	0.048 (p = 0.133)
F3-TP7	0.047 (-0.014 : 0.106)	0.491 (-0.014 : 0.106)	0.045 (p = 0.153)
C3-P4	0.038 (-0.019 : 0.090)	0.478 (-0.019 : 0.090)	0.038 (p = 0.184)
F4-T8	0.039 (-0.026 : 0.099)	0.438 (-0.026 : 0.099)	0.040 (p = 0.409)
C3-P3	0.035 (-0.029 : 0.100)	0.398 (-0.029 : 0.100)	0.035 (p = 0.288)
F4-P3	0.040 (-0.030 : 0.107)	0.356 (-0.030 : 0.107)	0.039 (p = 0.285)
C4-O2	0.029 (-0.014 : 0.077)	0.354 (-0.014 : 0.077)	0.029 (p = 0.261)
F3-P4	0.029 (-0.022 : 0.080)	0.338 (-0.022 : 0.080)	0.028 (p = 0.284)
C3-T8	-0.031 (-0.095 : 0.027)	-0.332 (-0.095 : 0.027)	-0.032 (p = 0.299)
C4-T8	0.027 (-0.024 : 0.079)	0.308 (-0.024 : 0.079)	0.027 (p = 0.342)
F4-O2	0.022 (-0.028 : 0.069)	0.262 (-0.028 : 0.069)	0.022 (p = 0.439)
C3-TP7	-0.022 (-0.077 : 0.030)	-0.258 (-0.077 : 0.030)	-0.021 (p = 0.425)
F3-T8	0.023 (-0.026 : 0.075)	0.252 (-0.026 : 0.075)	0.023 (p = 0.409)
C3-P8	0.021 (-0.040 : 0.080)	0.249 (-0.040 : 0.080)	0.021 (p = 0.483)
C3-O2	-0.020 (-0.073 : 0.033)	-0.235 (-0.073 : 0.033)	-0.019 (p = 0.466)
C4-T7	0.012 (-0.057 : 0.032)	-0.175 (-0.057 : 0.032)	-0.012 (p = 0.616)
F3-P3	0.020 (-0.039 : 0.085)	0.174 (-0.039 : 0.085)	0.019 (p = 0.568)
C4-P4	0.017 (-0.042 : 0.079)	0.174 (-0.042 : 0.079)	0.016 (p = 0.603)
C3-O1	-0.013 (-0.079 : 0.047)	-0.136 (-0.079 : 0.047)	-0.012 (p = 0.701)
C3-P7	0.008 (-0.053 : 0.064)	0.089 (-0.053 : 0.064)	-0.008 (p = 0.773)
F3-T7	0.004 (-0.064 : 0.070)	0.041 (-0.064 : 0.070)	-0.004 (p = 0.899)
C3-TP8	-0.005 (-0.077 : 0.062)	-0.035 (-0.077 : 0.062)	-0.005 (p = 0.868)
F3-O1	0.004 (-0.047 : 0.060)	0.032 (-0.047 : 0.060)	0.003 (p = 0.889)

(Continue) DELTA - HC vs DLB Bootstrapping and Permutation Results (Sensitivity Analysis and Validation)

**HC vs DLB**	**Mean Difference (Lower CI : Upper CI)**	**Effect Size (Lower Effect CI : Upper Effect CI)**	**Observed Difference (p-value)**
C3-T7	-0.085 (-0.162 : -0.012)	-1.084 (-0.162 : -0.012)	-0.084 **(p = 0.007*)**
C4-T7	-0.060 (-0.116 : -0.003)	-0.793 (-0.116 : -0.003)	-0.059 **(p = 0.048*)**
F4-T7	0.076 (0.012 : 0.139)	0.761 (0.012 : 0.139)	0.076 (p = 0.050)
F3-TP8	0.078 (0.017 : 0.141)	0.743 (0.017 : 0.141)	0.078 **(p = 0.045*)**
C4-P7	0.068 (0.012 : 0.127)	0.692 (0.012 : 0.127)	0.069 (p = 0.065)
C3-O2	0.047 (0.002 : 0.092)	0.662 (0.002 : 0.092)	0.046 (p = 0.091)
C4-P4	0.058 (0.005 : 0.111)	0.632 (0.005 : 0.111)	0.057 (p = 0.101)
C4-P3	0.065 (0.005 : 0.123)	0.607 (0.005 : 0.123)	0.065 (p = 0.106)
F4-TP8	0.077 (-0.015 : 0.167)	0.593 (-0.015 : 0.167)	0.076 (p = 0.129)
C3-P7	0.053 (-0.037 : 0.133)	0.580 (-0.037 : 0.133)	0.050 (p = 0.182)
F4-TP7	0.065 (0.001 : 0.133)	0.575 (0.001 : 0.133)	0.066 (p = 0.135)
F3-P7	0.067 (-0.010 : 0.146)	0.557 (-0.010 : 0.146)	0.069 (p = 0.137)
F4-P8	0.065 (-0.027 : 0.161)	0.510 (-0.027 : 0.161)	0.069 (p = 0.174)
F3-TP7	0.044 (-0.016 : 0.102)	0.441 (-0.016 : 0.102)	0.044 (p = 0.237)
C4-TP8	0.035 (-0.037 : 0.101)	0.399 (-0.037 : 0.101)	0.034 (p = 0.299)
C4-O1	0.035 (-0.026 : 0.093)	0.358 (-0.026 : 0.093)	0.034 (p = 0.337)
C3-P4	0.029 (-0.032 : 0.090)	0.348 (-0.032 : 0.090)	0.028 (p = 0.365)
C3-TP7	-0.025 (-0.096 : 0.038)	-0.334 (-0.096 : 0.038)	-0.025 (p = 0.391)
C3-TP8	0.028 (-0.039 : 0.087)	0.322 (-0.039 : 0.087)	0.027 (p = 0.414)
F3-P3	0.037 (-0.027 : 0.106)	0.317 (-0.027 : 0.106)	0.037 (p = 0.383)
F4-P7	0.043 (-0.021 : 0.115)	0.309 (-0.021 : 0.115)	0.042 (p = 0.407)
F3-P8	0.028 (-0.043 : 0.095)	0.287 (-0.043 : 0.095)	0.028 (p = 0.453)
C4-TP7	0.024 (-0.026 : 0.074)	0.287 (-0.026 : 0.074)	0.024 (p = 0.437)
F4-T8	0.029 (-0.049 : 0.102)	0.276 (-0.049 : 0.102)	0.029 (p = 0.462)
C3-P8	0.023 (-0.047 : 0.084)	0.271 (-0.047 : 0.084)	0.021 (p = 0.523)
F4-P4	0.026 (-0.058 : 0.110)	0.251 (-0.058 : 0.110)	0.025 (p = 0.528)
F3-O1	-0.019 (-0.079 : 0.038)	-0.242 (-0.079 : 0.039)	-0.020 (p = 0.535)
F3-T8	0.021 (-0.047 : 0.088)	0.218 (-0.047 : 0.088)	0.020 (p = 0.536)
F3-T7	0.015 (-0.038 : 0.064)	0.166 (-0.038 : 0.064)	0.014 (p = 0.652)
C4-P8	0.011 (-0.070 : 0.085)	0.157 (-0.070 : 0.085)	0.011 (p = 0.759)
C3-T8	0.013 (-0.043 : 0.066)	0.157 (-0.043 : 0.066)	0.013 (p = 0.659)
F4-O2	0.013 (-0.049 : 0.070)	0.140 (-0.049 : 0.070)	0.011 (p = 0.735)
C3-P3	-0.011 (-0.089 : 0.057)	-0.120 (-0.089 : 0.057)	-0.010 (p = 0.766)
F3-O2	-0.002 (-0.046 : 0.043)	-0.022 (-0.046 : 0.043)	-0.000 (p = 0.989)
F3-P4	-0.001 (-0.066 : 0.055)	-0.021 (-0.066 : 0.055)	0.000 (p = 1.000)
C3-O1	0.002 (-0.056 : 0.059)	0.017 (-0.056 : 0.059)	0.002 (p = 0.957)
C4-O2	0.001 (-0.067 : 0.069)	0.016 (-0.067 : 0.069)	0.003 (p = 0.915)
C4-T8	0.000 (-0.088 : 0.079)	0.015 (-0.088 : 0.079)	-0.000 (p = 0.987)
F4-O1	0.000 (-0.065 : 0.063)	-0.009 (-0.065 : 0.063)	-0.000 (p = 0.984)
F4-P3	0.021 (-0.044 : 0.086)	-0.009 (-0.044 : 0.086)	0.020 (p = 0.645)

Supplementary Table 1.2. THETA - HC vs PDMCI Bootstrapping and Permutation Results (Sensitivity Analysis and Validation)

**HC vs PDMCI**	**Mean Difference (Lower CI : Upper CI)**	**Effect Size (Lower Effect CI : Upper Effect CI)**	**Observed Difference (p-value)**
F3-T8	0.068 (0.020 : 0.119)	0.790 (0.020 : 0.119)	0.066 **(p = 0.048∗)**
C3-P7	-0.061 (-0.115 : -0.013)	-0.739 (-0.115 : -0.013)	-0.061 **(p = 0.048∗)**
F4-TP8	0.072 (0.007 : 0.135)	0.664 (0.007 : 0.135)	0.072 **(p = 0.048∗)**
F3-TP8	0.058 (0.004 : 0.115)	0.599 (0.004 : 0.115)	0.057 (p = 0.062)
C4-O1	0.039 (-0.011 : 0.084)	0.553 (-0.011 : 0.084)	0.038 (p = 0.099)
F4-T8	0.046 (-0.003 : 0.099)	0.536 (-0.003 : 0.099)	0.045 (p = 0.086)
F4-P8	0.057 (-0.006 : 0.122)	0.497 (-0.006 : 0.122)	0.059 **(p = 0.046∗)**
F3-P8	0.045 (-0.007 : 0.104)	0.478 (-0.007 : 0.104)	0.045 (p = 0.130)
C3-T7	-0.024 (-0.064 : 0.021)	-0.401 (-0.064 : 0.021)	-0.022 (p = 0.310)
F4-O2	0.032 (-0.019 : 0.078)	0.390 (-0.019 : 0.078)	0.031 (p = 0.228)
C4-O2	0.030 (-0.018 : 0.083)	0.353 (-0.018 : 0.083)	-0.053 (p = 0.032)
F4-O1	0.026 (-0.018 : 0.069)	0.345 (-0.083 : 0.069)	0.027 (p = 0.257)
C3-O1	-0.029 (-0.083 : 0.025)	-0.335 (-0.018 : 0.025)	-0.028 (p = 0.286)
F4-P7	0.035 (-0.034 : 0.101)	0.317 (-0.034 : 0.101)	0.034 (p = 0.325)
F3-TP7	0.032 (-0.029 : 0.091)	0.306 (-0.029 : 0.091)	0.031 (p = 0.328)
C4-TP7	0.024 (-0.024 : 0.068)	0.301 (-0.024 : 0.068)	0.024 (p = 0.323)
C4-T7	0.021 (-0.023 : 0.065)	0.288 (-0.023 : 0.065)	0.020 (p = 0.376)
F3-P4	0.017 (-0.024 : 0.054)	0.284 (-0.024 : 0.054)	0.016 (p = 0.415)
F3-P3	0.020 (-0.022 : 0.066)	0.279 (-0.022 : 0.066)	0.022 (p = 0.336)
C3-O2	0.020 (-0.021 : 0.061)	0.276 (-0.021 : 0.061)	0.020 (p = 0.373)
F3-O1	0.022 (-0.024 : 0.068)	0.270 (-0.024 : 0.068)	0.022 (p = 0.379)
F4-TP7	0.027 (-0.038 : 0.094)	0.245 (-0.038 : 0.094)	0.027 (p = 0.401)
F4-P4	0.018 (-0.028 : 0.061)	0.244 (-0.028 : 0.061)	0.018 (p = 0.443)
C3-TP8	0.016 (-0.025 : 0.059)	0.237 (-0.025 : 0.059)	-0.053 (p = 0.032)
F3-O2	0.019 (-0.034 : 0.068)	0.231 (-0.034 : 0.068)	0.019 (p = 0.465)
C3-P4	0.016 (-0.023 : 0.058)	0.211 (-0.023 : 0.058)	0.016 (p = 0.465)
C3-P3	-0.023 (-0.092 : 0.041)	-0.194 (-0.092 : 0.041)	-0.023 (p = 0.533)
C4-TP8	0.012 (-0.027 : 0.052)	0.192 (-0.027 : 0.052)	0.010 (p = 0.608)
C3-T8	-0.011 (-0.051 : 0.029)	-0.163 (-0.051 : 0.029)	-0.010 (p = 0.599)
C3-TP7	-0.010 (-0.053 : 0.034)	-0.157 (-0.053 : 0.034)	-0.010 (p = 0.629)
F4-T7	0.013 (-0.048 : 0.070)	0.129 (-0.048 : 0.070)	0.013 (p = 0.677)
C4-P4	0.009 (-0.034 : 0.057)	0.127 (-0.044 : 0.057)	0.009 (p = 0.724)
C4-P3	0.010 (-0.038 : 0.063)	0.124 (-0.038 : 0.063)	0.010 (p = 0.695)
C3-P8	0.007 (-0.045 : 0.056)	0.107 (-0.045 : 0.056)	0.006 (p = 0.803)
C4-P8	-0.011 (-0.072 : 0.043)	-0.107 (-0.072 : 0.043)	-0.010 (p = 0.717)
F3-T7	0.009 (-0.046 : 0.064)	0.107 (-0.046 : 0.064)	0.009 (p = 0.732)
C4-P7	0.006 (-0.046 : 0.051)	0.083 (-0.046 : 0.051)	0.007 (p = 0.767)
C4-T8	-0.002 (-0.041 : 0.037)	-0.025 (-0.041 : 0.037)	-0.001 (p = 0.941)
F4-P3	0.003 (-0.040 : 0.049)	0.025 (-0.040 : 0.049)	0.003 (p = 0.891)
F3-P7	-0.002 (-0.065 : 0.070)	-0.022 (-0.065 : 0.070)	-0.002 (p = 0.944)

(Continue) THETA - HC vs PDD Bootstrapping and Permutation Results (Sensitivity Analysis and Validation)

**HC vs PDD**	**Mean Difference (Lower CI : Upper CI)**	**Effect Size (Lower Effect CI : Upper Effect CI)**	**Observed Difference (p-value)**
F4-P8	0.102 (0.022 : 0.174)	0.854 (0.022 : 0.174)	0.102 **(p = 0.012∗)**
F4-O2	0.066 (0.015 : 0.115)	0.820 (0.015 : 0.115)	0.067 **(p = 0.011∗)**
F4-TP8	0.094 (0.015 : 0.175)	0.757 (0.015 : 0.175)	0.093 **(p = 0.026∗)**
C3-TP8	0.034 (-0.006 : 0.073)	0.524 (-0.006 : 0.073)	0.033 (p = 0.124)
F3-TP7	0.054 (-0.008 : 0.117)	0.524 (-0.008 : 0.117)	0.053 (p = 0.116)
C4-T7	0.034 (-0.004 : 0.070)	0.509 (-0.004 : 0.070)	0.033 (p = 0.123)
F3-O1	0.041 (-0.007 : 0.086)	0.505 (-0.007 : 0.086)	0.041 (p = 0.123)
C4-TP7	0.038 (-0.006 : 0.081)	0.504 (-0.006 : 0.081)	0.038 (p = 0.118)
F4-T7	0.047 (-0.011 : 0.106)	0.495 (-0.011 : 0.106)	0.045 (p = 0.142)
C3-O2	0.031 (-0.009 : 0.072)	0.464 (-0.009 : 0.072)	0.031 (p = 0.142)
F4-P7	0.046 (-0.010 : 0.107)	0.440 (-0.010 : 0.107)	0.044 (p = 0.179)
F4-TP7	0.043 (-0.012 : 0.101)	0.413 (-0.012 : 0.101)	0.042 (p = 0.206)
F3-T8	0.039 (-0.033 : 0.109)	0.385 (-0.033 : 0.109)	0.040 (**(p = 0.028∗)**
F3-P7	0.038 (-0.021 : 0.096)	0.381 (-0.021 : 0.096)	0.038 (p = 0.242)
C3-TP7	0.023 (-0.018 : 0.068)	0.312 (-0.018 : 0.068)	0.022 (p = 0.330)
C4-P7	0.023 (-0.023 : 0.070)	0.310 (-0.023 : 0.070)	0.022 (p = 0.350)
F3-TP8	0.029 (-0.047 : 0.094)	0.279 (-0.047 : 0.094)	0.027 (p = 0.454)
C3-T8	-0.017 (-0.056 : 0.022)	-0.277 (-0.056 : 0.022)	-0.017 (p = 0.382)
F4-T8	0.027 (-0.062 : 0.100)	0.272 (-0.062 : 0.100)	0.025 (p = 0.543)
C4-P3	-0.021 (-0.072 : 0.027)	-0.259 (-0.072 : 0.027)	-0.020 (p = 0.446)
C3-P3	-0.022 (-0.087 : 0.044)	-0.226 (-0.087 : 0.044)	-0.022 (p = 0.494)
F3-P3	-0.015 (-0.065 : 0.034)	-0.185 (-0.065 : 0.034)	-0.014 (p = 0.583)
F3-P8	0.017 (-0.056 : 0.086)	0.174 (-0.056 : 0.086)	0.018 (p = 0.611)
C4-TP8	0.015 (-0.036 : 0.059)	0.170 (-0.036 : 0.059)	0.012 (p = 0.573)
F3-O2	-0.013 (-0.062 : 0.034)	-0.168 (-0.062 : 0.034)	-0.013 (p = 0.597)
C3-P7	-0.008 (-0.049 : 0.034)	-0.120 (-0.049 : 0.034)	-0.008 (p = 0.723)
F4-O1	-0.007 (-0.051 : 0.041)	-0.099 (-0.051 : 0.041)	-0.006 (p = 0.771)
F3-T7	0.009 (-0.042 : 0.063)	0.098 (-0.042 : 0.063)	0.009 (p = 0.736)
C3-T7	0.008 (-0.035 : 0.055)	0.095 (-0.035 : 0.055)	0.009 (p = 0.731)
C4-O1	0.005 (-0.033 : 0.042)	0.093 (-0.033 : 0.042)	0.004 (p = 0.830)
F3-P4	0.006 (-0.030 : 0.042)	0.092 (-0.030 : 0.042)	0.005 (p = 0.769)
C4-T8	0.006 (-0.035 : 0.046)	0.088 (-0.035 : 0.046)	0.006 (p = 0.793)
F4-P4	0.007 (-0.041 : 0.058)	0.084 (-0.041 : 0.058)	0.007 (p = 0.732)
C3-P4	0.007 (-0.034 : 0.051)	0.079 (-0.034 : 0.051)	0.007 (p = 0.775)
C3-O1	0.005 (-0.041 : 0.051)	0.056 (-0.041 : 0.051)	0.004 (p = 0.851)
C4-P4	-0.006 (-0.064 : 0.048)	-0.055 (-0.064 : 0.048)	-0.003 (p = 0.901)
C3-P8	-0.003 (-0.051 : 0.044)	-0.051 (-0.051 : 0.044)	-0.002 (p = 0.912)
C4-P8	-0.002 (-0.039 : 0.037)	0.033 (-0.039 : 0.037)	-0.001 (p = 0.932)
F4-P3	0.002 (-0.040 : 0.045)	0.017 (-0.040 : 0.045)	0.001 (p = 0.932)
C4-O2	-0.001 (-0.054 : 0.050)	-0.010 (-0.054 : 0.050)	-0.000 (p = 0.997)

(Continue) THETA - HC vs DLB Bootstrapping and Permutation Results (Sensitivity Analysis and Validation)

**HC vs DLB**	**Mean Difference (Lower CI : Upper CI)**	**Effect Size (Lower Effect CI : Upper Effect CI)**	**Observed Difference (p-value)**
C3-T7	-0.067 (-0.156 : 0.009)	-0.692 (-0.156 : 0.009)	-0.064 (p = 0.090)
C4-TP7	0.052 (0.006 : 0.095)	0.650 (0.006 : 0.095)	0.051 (p = 0.083)
C3-P7	-0.048 (-0.116 : 0.018)	-0.628 (-0.116 : 0.018)	-0.050 (p = 0.116)
F4-P8	0.067 (-0.011 : 0.136)	0.557 (-0.011 : 0.136)	0.069 (**(p = 0.049**$*$**)**
F3-TP8	0.056 (-0.008 : 0.124)	0.539 (-0.008 : 0.124)	0.057 (p = 0.142)
C4-P7	0.039 (-0.009 : 0.085)	0.534 (-0.009 : 0.085)	0.040 (p = 0.161)
F4-T8	-0.064 (-0.169 : 0.035)	-0.521 (-0.169 : 0.035)	-0.063 (p = 0.174)
C4-T8	-0.041 (-0.115 : 0.016)	-0.474 (-0.115 : 0.016)	-0.042 (p = 0.184)
F4-P7	0.051 (-0.031 : 0.124)	0.453 (-0.031 : 0.124)	0.050 (p = 0.262)
C3-P3	-0.046 (-0.137 : 0.038)	-0.452 (-0.137 : 0.038)	-0.044 (p = 0.259)
F4-TP8	0.053 (-0.012 : 0.123)	0.449 (-0.012 : 0.123)	0.053 (p = 0.232)
F3-P3	-0.039 (-0.120 : 0.030)	-0.408 (-0.120 : 0.030)	-0.038 (p = 0.276)
C4-P4	-0.045 (-0.157 : 0.037)	-0.382 (-0.157 : 0.037)	-0.046 (p = 0.250)
F4-TP7	0.041 (-0.017 : 0.100)	0.378 (-0.017 : 0.100)	0.040 (p = 0.327)
C4-P8	-0.048 (-0.166 : 0.040)	-0.375 (-0.166 : 0.040)	-0.048 (p = 0.266)
F4-P4	-0.033 (-0.109 : 0.036)	-0.367 (-0.109 : 0.036)	-0.033 (p = 0.333)
F3-O1	0.030 (-0.017 : 0.080)	0.363 (-0.017 : 0.080)	0.029 (p = 0.347)
F3-T7	0.033 (-0.028 : 0.094)	0.359 (-0.028 : 0.094)	0.032 (p = 0.361)
C3-O2	0.024 (-0.029 : 0.073)	0.327 (-0.029 : 0.073)	0.022 (p = 0.432)
F3-P8	0.029 (-0.034 : 0.095)	0.282 (-0.034 : 0.095)	0.030 (p = 0.414)
C4-O1	0.015 (-0.066 : 0.081)	0.268 (-0.066 : 0.081)	0.011 (p = 0.742)
C4-O2	0.022 (-0.054 : 0.097)	0.259 (-0.054 : 0.097)	0.021 (p = 0.543)
C4-TP8	-0.025 (-0.120 : 0.063)	-0.235 (-0.120 : 0.063)	-0.025 (p = 0.478)
F4-O2	0.019 (-0.046 : 0.080)	0.227 (-0.046 : 0.080)	-0.018 (**(p = 0.045**$*$**)**
C4-T7	-0.018 (-0.083 : 0.046)	-0.205 (-0.083 : 0.046)	-0.017 (p = 0.560)
C3-P8	-0.014 (-0.062 : 0.037)	-0.205 (-0.062 : 0.037)	-0.014 (p = 0.613)
F3-P7	0.021 (-0.064 : 0.092)	0.200 (-0.064 : 0.092)	0.020 (p = 0.619)
C3-TP7	-0.016 (-0.079 : 0.048)	-0.194 (-0.079 : 0.048)	-0.016 (p = 0.608)
F3-P4	0.010 (-0.041 : 0.056)	0.167 (-0.041 : 0.056)	0.009 (p = 0.710)
F4-O1	-0.017 (-0.093 : 0.051)	-0.165 (-0.093 : 0.051)	-0.016 (p = 0.642)
C3-O1	-0.012 (-0.071 : 0.043)	-0.164 (-0.071 : 0.043)	-0.011 (p = 0.692)
C3-TP8	0.011 (-0.044 : 0.058)	0.156 (-0.044 : 0.058)	0.011 (p = 0.655)
F3-O2	0.014 (-0.054 : 0.085)	0.155 (-0.054 : 0.085)	0.015 (p = 0.670)
C3-T8	-0.016 (-0.097 : 0.051)	-0.153 (-0.097 : 0.051)	-0.017 (p = 0.583)
F3-TP7	0.017 (-0.059 : 0.093)	0.151 (-0.059 : 0.093)	0.016 (p = 0.686)
F4-T7	0.009 (-0.106 : 0.097)	0.131 (-0.106 : 0.097)	0.008 (p = 0.851)
C3-P4	0.008 (-0.043 : 0.058)	0.080 (-0.043 : 0.058)	0.008 (p = 0.767)
F3-T8	0.003 (-0.086 : 0.091)	0.042 (-0.086 : 0.091)	0.002 (p = 0.949)
C4-P3	0.002 (-0.060 : 0.057)	0.025 (-0.060 : 0.057)	0.003 (p = 0.915)
F4-P3	-0.026 (-0.115 : 0.053)	0.045 (-0.115 : 0.053)	-0.027 (p = 0.467)
