# Supplementary material for: Wavelet event-related EEG phase coherence as a discriminant biomarker of the cognitive status in Parkinson’s and Lewy body disease
Source: Front Hum Neurosci. 2026 Apr 2;20:1696861. doi: 10.3389/fnhum.2026.1696861 (PMC13083073; doi:10.3389/fnhum.2026.1696861)
Supplement: Supplementary file 2 [file Table_2.pdf]

Supplementary Table 2.1. DELTA - Weighted Coherence Values for MMSE and Error Scores (Mean ± SD)

Electrode Pairs	HC		PD-MCI		PDD		DLB	
	**MMSE**	**Error Scores**	**MMSE**	**Error Scores**	**MMSE**	**Error Scores**	**MMSE**	**Error Scores**
C3_O1	0.7 (0.3)	0.7 (0.2)	0.8 (0.2)	0.7 (0.2)	0.8 (0.2)	0.9 (0.8)	0.7 (0.2)	0.7 (0.3)
C3_O2	0.7 (0.2)	0.7 (0.4)	0.7 (0.2)	0.7 (0.4)	0.8 (0.2)	0.9 (0.8)	0.9 (0.2)	0.8 (0.2)
C3_P3	0.9 (0.2)	0.9 (0.4)	0.9 (0.3)	1.0 (0.6)	0.8 (0.3)	0.7 (0.3)	0.8 (0.1)	0.7 (0.2)
C3_P4	0.8 (0.2)	0.8 (0.3)	0.8 (0.2)	0.8 (0.4)	0.7 (0.2)	0.8 (0.6)	0.7 (0.1)	0.9 (0.6)
C3_P7	0.8 (0.2)	0.7 (0.2)	0.8 (0.3)	0.7 (0.3)	0.8 (0.2)	0.9 (0.5)	0.8 (0.1)	0.8 (0.4)
C3_P8	0.8 (0.2)	0.8 (0.4)	0.8 (0.3)	0.8 (0.6)	0.7 (0.3)	0.8 (0.7)	0.7 (0.2)	0.7 (0.1)
C3_T7	0.6 (0.2)	0.7 (0.4)	0.8 (0.3)	0.9 (0.4)	0.7 (0.2)	0.6 (0.2)	0.7 (0.1)	0.6 (0.1)
C3_T8	0.7 (0.2)	0.7 (0.5)	0.7 (0.3)	0.7 (0.3)	0.7 (0.3)	0.7 (0.2)	0.7 (0.1)	0.8 (0.5)
C3_TP7	0.7 (0.2)	0.7 (0.4)	0.8 (0.3)	0.7 (0.2)	0.7 (0.2)	0.8 (0.5)	0.6 (0.1)	0.8 (0.4)
C3_TP8	0.7 (0.2)	0.7 (0.2)	0.7 (0.2)	0.6 (0.3)	0.7 (0.3)	0.9 (0.8)	0.8 (0.2)	0.9 (0.2)
C4_O1	0.8 (0.3)	0.8 (0.3)	0.7 (0.2)	0.7 (0.3)	0.7 (0.1)	0.7 (0.3)	0.7 (0.2)	0.7 (0.2)
C4_O2	0.8 (0.2)	0.8 (0.4)	0.8 (0.2)	0.9 (0.5)	0.7 (0.2)	0.6 (0.1)	0.7 (0.2)	0.7 (0.2)
C4_P3	0.9 (0.3)	0.8 (0.5)	0.7 (0.2)	0.7 (0.2)	0.7 (0.2)	0.7 (0.5)	0.8 (0.3)	1.0 (1.0)
C4_P4	0.8 (0.3)	0.7 (0.2)	0.7 (0.2)	0.7 (0.3)	0.8 (0.2)	0.8 (0.4)	0.7 (0.2)	0.7 (0.5)
C4_P7	0.8 (0.3)	0.8 (0.7)	0.7 (0.2)	0.8 (0.4)	0.6 (0.2)	0.6 (0.3)	0.7 (0.2)	0.6 (0.1)
C4_P8	0.8 (0.2)	0.9 (0.6)	0.8 (0.3)	0.9 (0.6)	0.7 (0.2)	0.6 (0.2)	0.7 (0.2)	0.6 (0.2)
C4_T7	0.6 (0.2)	0.7 (0.3)	0.7 (0.2)	0.8 (0.5)	0.7 (0.2)	0.6 (0.1)	0.6 (0.1)	0.5 (0.1)
C4_T8	0.8 (0.2)	0.7 (0.2)	0.7 (0.3)	0.7 (0.4)	0.7 (0.2)	0.6 (0.1)	0.6 (0.2)	0.7 (0.4)
C4_TP7	0.7 (0.2)	0.6 (0.2)	0.7 (0.2)	0.6 (0.2)	0.6 (0.1)	0.7 (0.3)	0.5 (0.1)	0.6 (0.5)
C4_TP8	0.9 (0.2)	0.8 (0.3)	0.8 (0.2)	0.8 (0.6)	0.7 (0.2)	0.6 (0.4)	0.7 (0.2)	0.7 (0.2)
F3_O1	0.7 (0.2)	0.8 (0.3)	0.8 (0.2)	0.9 (0.7)	0.7 (0.2)	0.7 (0.2)	0.7 (0.2)	0.6 (0.2)
F3_O2	0.8 (0.2)	0.8 (0.4)	0.8 (0.2)	0.8 (0.4)	0.7 (0.2)	0.7 (0.3)	0.7 (0.1)	0.8 (0.6)
F3_P3	0.8 (0.3)	0.8 (0.3)	0.7 (0.2)	0.7 (0.2)	0.7 (0.2)	0.8 (0.6)	0.7 (0.2)	0.8 (0.4)
F3_P4	0.7 (0.2)	0.7 (0.3)	0.7 (0.2)	0.7 (0.3)	0.7 (0.2)	0.7 (0.3)	0.7 (0.2)	0.7 (0.2)
F3_P7	0.9 (0.3)	1.0 (0.5)	0.8 (0.2)	0.8 (0.4)	0.7 (0.2)	0.6 (0.2)	0.8 (0.2)	0.8 (0.2)
F3_P8	0.9 (0.3)	0.9 (0.3)	0.8 (0.2)	0.9 (0.6)	0.7 (0.2)	0.6 (0.2)	0.7 (0.1)	0.8 (0.1)
F3_T7	0.7 (0.2)	0.7 (0.3)	0.7 (0.2)	0.7 (0.3)	0.7 (0.3)	0.7 (0.3)	0.8 (0.2)	0.8 (0.4)
F3_T8	0.8 (0.2)	0.7 (0.2)	0.7 (0.2)	0.7 (0.2)	0.7 (0.2)	0.8 (0.4)	0.8 (0.2)	0.8 (0.6)
F3_TP7	0.8 (0.3)	0.8 (0.4)	0.8 (0.2)	0.7 (0.3)	0.7 (0.2)	0.7 (0.2)	0.8 (0.2)	0.8 (0.2)
F3_TP8	0.8 (0.2)	1.0 (0.6)	0.7 (0.2)	0.8 (0.5)	0.7 (0.2)	0.6 (0.2)	0.8 (0.2)	0.7 (0.1)
F4_O1	0.9 (0.3)	0.8 (0.5)	0.8 (0.3)	0.8 (0.3)	0.7 (0.2)	0.6 (0.1)	0.7 (0.2)	0.8 (0.4)
F4_O2	0.8 (0.3)	0.8 (0.4)	0.7 (0.2)	0.7 (0.2)	0.7 (0.2)	0.8 (0.5)	0.7 (0.3)	0.7 (0.2)
F4_P3	0.8 (0.2)	0.7 (0.3)	0.7 (0.1)	0.6 (0.1)	0.7 (0.2)	0.8 (0.6)	0.7 (0.2)	0.6 (0.2)
F4_P4	0.8 (0.3)	0.9 (0.5)	0.8 (0.2)	0.8 (0.3)	0.7 (0.2)	0.7 (0.4)	0.7 (0.2)	0.7 (0.6)
F4_P7	0.9 (0.4)	0.9 (0.5)	0.8 (0.2)	0.9 (0.6)	0.7 (0.2)	0.6 (0.2)	0.8 (0.2)	0.7 (0.6)
F4_P8	1.0 (0.3)	1.0 (0.6)	0.8 (0.3)	0.8 (0.4)	0.7 (0.2)	0.7 (0.3)	0.8 (0.3)	0.9 (0.6)
F4_T7	0.8 (0.3)	0.8 (0.3)	0.8 (0.2)	0.6 (0.2)	0.6 (0.2)	0.7 (0.3)	0.8 (0.1)	0.9 (0.2)
F4_T8	0.8 (0.3)	0.8 (0.3)	0.7 (0.2)	0.8 (0.3)	0.7 (0.2)	0.7 (0.3)	0.8 (0.2)	0.7 (0.1)
F4_TP7	0.8 (0.3)	0.9 (0.8)	0.7 (0.2)	0.8 (0.6)	0.8 (0.2)	0.6 (0.2)	0.6 (0.2)	0.6 (0.8)
F4_TP8	1.0 (0.3)	1.2 (1.0)	0.8 (0.3)	0.8 (0.4)	0.7 (0.2)	0.6 (0.2)	0.8 (0.2)	0.9 (0.8)

∗ Values reflect mean ± SD of weighted coherence for selected electrode pairs across groups. MMSE and error scores are cognitive performance measures. Pairs were selected based on relevance in classification tasks.

Supplementary Table 2.2. THETA - Weighted Coherence Values for MMSE and Error Scores (Mean ± SD)

**Electrode Pairs**	**HC**		**PD-MCI**		**PDD**		**DLB**	
	**MMSE**	**Error Scores**	**MMSE**	**Error Scores**	**MMSE**	**Error Scores**	**MMSE**	**Error Scores**
C3_O1	0.7 (0.2)	0.7 (0.3)	0.7 (0.2)	0.7 (0.2)	0.7 (0.2)	0.8 (0.5)	0.7 (0.2)	0.7 (0.2)
C3_O2	0.7 (0.2)	0.7 (0.4)	0.6 (0.2)	0.6 (0.2)	0.6 (0.1)	0.6 (0.2)	0.7 (0.2)	0.7 (0.5)
C3_P3	0.7 (0.2)	0.8 (0.7)	0.9 (0.3)	0.9 (0.6)	0.8 (0.3)	0.7 (0.3)	0.7 (0.2)	0.6 (0.2)
C3_P4	0.7 (0.2)	0.6 (0.2)	0.7 (0.1)	0.6 (0.1)	0.7 (0.1)	0.7 (0.2)	0.6 (0.1)	0.7 (0.4)
C3_P7	0.7 (0.2)	0.7 (0.5)	0.8 (0.3)	0.9 (0.6)	0.7 (0.1)	0.7 (0.4)	0.8 (0.2)	0.7 (0.2)
C3_P8	0.7 (0.2)	0.7 (0.3)	0.7 (0.2)	0.7 (0.3)	0.7 (0.2)	0.6 (0.1)	0.6 (0.2)	0.6 (0.2)
C3_T7	0.6 (0.2)	0.6 (0.3)	0.8 (0.3)	0.7 (0.2)	0.6 (0.2)	0.7 (0.3)	0.7 (0.1)	0.8 (0.5)
C3_T8	0.6 (0.1)	0.7 (0.3)	0.7 (0.3)	0.8 (0.5)	0.7 (0.2)	0.6 (0.1)	0.8 (0.2)	0.6 (0.2)
C3_TP7	0.7 (0.2)	0.6 (0.2)	0.7 (0.2)	0.6 (0.2)	0.6 (0.1)	0.7 (0.4)	0.7 (0.2)	0.8 (0.5)
C3_TP8	0.7 (0.2)	0.8 (0.5)	0.7 (0.2)	0.8 (0.5)	0.6 (0.2)	0.5 (0.1)	0.6 (0.2)	0.6 (0.2)
C4_O1	0.7 (0.2)	0.6 (0.1)	0.7 (0.3)	0.6 (0.2)	0.7 (0.1)	0.8 (0.5)	0.6 (0.2)	0.7 (0.4)
C4_O2	0.7 (0.2)	0.8 (0.4)	0.6 (0.3)	0.6 (0.4)	0.7 (0.2)	0.7 (0.3)	0.7 (0.2)	0.6 (0.2)
C4_P3	0.8 (0.2)	0.6 (0.2)	0.6 (0.2)	0.6 (0.2)	0.7 (0.2)	0.9 (0.8)	0.6 (0.2)	0.6 (0.2)
C4_P4	0.6 (0.2)	0.8 (0.6)	0.8 (0.3)	1.0 (1.1)	0.8 (0.2)	0.7 (0.2)	0.7 (0.2)	0.6 (0.2)
C4_P7	0.7 (0.2)	0.7 (0.4)	0.7 (0.2)	0.7 (0.4)	0.7 (0.2)	0.6 (0.2)	0.6 (0.1)	0.6 (0.1)
C4_P8	0.7 (0.2)	0.7 (0.3)	0.8 (0.4)	0.8 (0.4)	0.7 (0.1)	0.6 (0.1)	0.6 (0.2)	0.7 (0.3)
C4_T7	0.7 (0.2)	0.7 (0.5)	0.7 (0.2)	0.7 (0.3)	0.6 (0.1)	0.5 (0.1)	0.6 (0.1)	0.6 (0.1)
C4_T8	0.7 (0.2)	0.6 (0.2)	0.8 (0.2)	0.7 (0.2)	0.7 (0.2)	0.8 (0.4)	0.7 (0.2)	0.9 (0.6)
C4_TP7	0.7 (0.2)	0.7 (0.3)	0.6 (0.1)	0.7 (0.5)	0.6 (0.1)	0.6 (0.3)	0.6 (0.1)	0.7 (0.4)
C4_TP8	0.7 (0.2)	0.7 (0.1)	0.8 (0.3)	0.7 (0.3)	0.7 (0.2)	0.8 (0.5)	0.7 (0.1)	0.6 (0.1)
F3_O1	0.7 (0.2)	0.8 (0.7)	0.7 (0.2)	0.8 (0.5)	0.6 (0.2)	0.6 (0.1)	0.7 (0.1)	0.6 (0.1)
F3_O2	0.7 (0.2)	0.6 (0.2)	0.7 (0.2)	0.6 (0.2)	0.7 (0.2)	0.8 (0.5)	0.6 (0.2)	0.7 (0.4)
F3_P3	0.7 (0.2)	0.7 (0.3)	0.7 (0.2)	0.8 (0.6)	0.7 (0.2)	0.6 (0.2)	0.6 (0.1)	0.5 (0.1)
F3_P4	0.7 (0.2)	0.6 (0.2)	0.8 (0.2)	0.6 (0.3)	0.7 (0.1)	0.6 (0.2)	0.6 (0.1)	0.5 (0.1)
F3_P7	0.8 (0.3)	0.8 (0.3)	0.8 (0.2)	0.7 (0.2)	0.7 (0.2)	0.7 (0.3)	0.8 (0.3)	1.1 (0.9)
F3_P8	0.8 (0.3)	0.8 (0.5)	0.7 (0.2)	0.8 (0.4)	0.7 (0.3)	0.7 (0.2)	0.6 (0.1)	0.5 (0.1)
F3_T7	0.7 (0.2)	0.7 (0.2)	0.7 (0.2)	0.6 (0.2)	0.7 (0.2)	0.8 (0.4)	0.6 (0.1)	0.8 (0.5)
F3_T8	0.8 (0.2)	0.8 (0.3)	0.7 (0.3)	0.8 (0.6)	0.7 (0.3)	0.6 (0.3)	0.8 (0.3)	0.6 (0.2)
F3_TP7	0.8 (0.3)	0.9 (0.6)	0.8 (0.2)	0.8 (0.4)	0.7 (0.2)	0.8 (0.6)	0.8 (0.3)	0.7 (0.2)
F3_TP8	0.8 (0.3)	0.8 (0.5)	0.7 (0.2)	0.6 (0.2)	0.7 (0.3)	0.7 (0.3)	0.6 (0.2)	0.8 (0.5)
F4_O1	0.7 (0.2)	0.6 (0.2)	0.7 (0.3)	0.6 (0.2)	0.7 (0.2)	0.8 (0.3)	0.7 (0.2)	0.6 (0.1)
F4_O2	0.8 (0.2)	0.8 (0.3)	0.7 (0.2)	0.8 (0.4)	0.6 (0.2)	0.6 (0.2)	0.7 (0.1)	0.6 (0.2)
F4_P3	0.7 (0.2)	0.7 (0.4)	0.7 (0.3)	0.7 (0.2)	0.7 (0.1)	0.7 (0.3)	0.7 (0.2)	0.6 (0.1)
F4_P4	0.7 (0.2)	0.8 (0.5)	0.7 (0.2)	0.7 (0.4)	0.7 (0.2)	0.6 (0.2)	0.7 (0.1)	0.5 (0.1)
F4_P7	0.8 (0.3)	0.8 (0.3)	0.7 (0.2)	0.7 (0.4)	0.7 (0.2)	0.6 (0.2)	0.8 (0.2)	0.7 (0.4)
F4_P8	0.9 (0.3)	0.8 (0.3)	0.8 (0.2)	0.8 (0.5)	0.7 (0.3)	0.8 (0.7)	0.8 (0.2)	0.7 (0.2)
F4_T7	0.8 (0.3)	0.8 (0.5)	0.7 (0.3)	0.7 (0.2)	0.6 (0.2)	0.6 (0.2)	0.8 (0.2)	1.0 (0.7)
F4_T8	0.8 (0.3)	0.7 (0.2)	0.8 (0.3)	0.7 (0.3)	0.7 (0.4)	0.7 (0.4)	0.7 (0.2)	0.8 (0.4)
F4_TP7	0.8 (0.3)	0.7 (0.3)	0.7 (0.2)	0.6 (0.2)	0.7 (0.2)	0.7 (0.4)	0.8 (0.2)	0.6 (0.2)
F4_TP8	0.9 (0.3)	0.9 (0.4)	0.8 (0.2)	0.5 (0.5)	0.7 (0.3)	0.6 (0.3)	0.8 (0.2)	0.8 (0.5)
