# Supplementary material for: Wavelet event-related EEG phase coherence as a discriminant biomarker of the cognitive status in Parkinson’s and Lewy body disease
Source: Front Hum Neurosci. 2026 Apr 2;20:1696861. doi: 10.3389/fnhum.2026.1696861 (PMC13083073; doi:10.3389/fnhum.2026.1696861)
Supplement: Supplementary file 3 [file Table_3.pdf]

Supplementary Table 3.1 DELTA – AUC Values For Inter- and Intrahemispheric Pairs Across Comparisons

**Electrode Pair Comparison**	**AUC Mean ± SD**
**HC vs PD-MCI**	
**Interhemispheric Pairs**	
C3_O2	0.3833 ± 0.2263
C3_P4	0.5833 ± 0.1804
C3_P8	0.6305 ± 0.2616
C3_T8	0.6222 ± 0.1838
C3_TP8	0.5611 ± 0.2134
C4_O1	0.5319 ± 0.2549
C4_P3	0.6166 ± 0.2301
C4_P7	0.6861 ± 0.2033
***C4_T7**	0.7239 ± 0.2841
C4_TP7	0.5750 ± 0.1981
***F3_O2**	0.7333 ± 0.2492
F3_P4	0.4472 ± 0.2430
***F3_P8**	0.7402 ± 0.2118
F3_T8	0.2833 ± 0.1864
***F3_TP8**	0.7055 ± 0.1743
F4_O1	0.6000 ± 0.2321
F4_P3	0.5916 ± 0.2467
F4_P7	0.6027 ± 0.1968
***F4_T7**	0.7944 ± 0.2635
F4_TP7	0.6277 ± 0.2928
**Intrahemispheric Pairs**	
C3_O1	0.5819 ± 0.2277
C3_P3	0.6333 ± 0.2956
C3_P7	0.6333 ± 0.2237
***C3_T7**	0.7833 ± 0.3236
C3_TP7	0.5027 ± 0.2917
C4_O2	0.5833 ± 0.3153
C4_P4	0.6875 ± 0.2138
C4_P8	0.6361 ± 0.2646
C4_T8	0.5750 ± 0.2625
C4_TP8	0.6694 ± 0.3018
F3_O1	0.4152 ± 0.3163
F3_P3	0.5861 ± 0.2227
F3_P7	0.6333 ± 0.2427
F3_T7	0.5555 ± 0.2622
F3_TP7	0.5750 ± 0.2074
F4_O2	0.6595 ± 0.1545
F4_P4	0.6194 ± 0.2625
F4_P8	0.7416 ± 0.1256
F4_T8	0.5611 ± 0.1846
F4_TP8	0.7000 ± 0.1731
**HC vs PDD**	
**Interhemispheric Pairs**	
C3_O2	0.6848 ± 0.1354
C3_P4	0.6750 ± 0.3004
C3_P8	0.7347 ± 0.2196
C3_T8	0.5583 ± 0.1771
C3_TP8	0.6383 ± 0.2180
C4_O1	0.8866 ± 0.1549
C4_P3	0.7416 ± 0.1644
C4_P7	0.8416 ± 0.2128
***C4_T7**	0.7558 ± 0.1745
C4_TP7	0.7200 ± 0.1880
***F3_O2**	0.8633 ± 0.1692
F3_P4	0.7458 ± 0.1839
***F3_P8**	0.9291 ± 0.1131
F3_T8	0.6388 ± 0.1907
***F3_TP8**	0.9866 ± 0.1062
F4_O1	0.7916 ± 0.1601
F4_P3	0.8116 ± 0.2359
F4_P7	0.9058 ± 0.0946
***F4_T7**	0.8275 ± 0.1479
F4_TP7	0.8750 ± 0.1080
**Intrahemispheric Pairs**	
C3_O1	0.8050 ± 0.0638
C3_P3	0.9083 ± 0.1155
C3_P7	0.8000 ± 0.2023
***C3_T7**	0.8863 ± 0.1583
C3_TP7	0.5287 ± 0.2704
C4_O2	0.7475 ± 0.2488
C4_P4	0.7458 ± 0.1629
C4_P8	0.8758 ± 0.1467
C4_T8	0.6916 ± 0.2251
C4_TP8	0.6716 ± 0.1441
F3_O1	0.5955 ± 0.1707
F3_P3	0.7400 ± 0.1860
F3_P7	0.8175 ± 0.1761
F3_T7	0.5508 ± 0.1732
F3_TP7	0.6591 ± 0.1501
F4_O2	0.7350 ± 0.2110
F4_P4	0.8466 ± 0.1731
F4_P8	0.8800 ± 0.1085
F4_T8	0.6900 ± 0.2131
F4_TP8	0.8041 ± 0.1023
**HC vs DLB**	
**Interhemispheric Pairs**	
C3_O2	0.8000 ± 0.1531
C3_P4	0.7000 ± 0.2698
C3_P8	0.8000 ± 0.2048
C3_T8	0.5750 ± 0.2734
C3_TP8	0.6333 ± 0.2048
C4_O1	0.6000 ± 0.2249
C4_P3	0.6500 ± 0.3552
C4_P7	0.7333 ± 0.3351
***C4_T7**	0.7916 ± 0.1926
C4_TP7	0.5833 ± 0.2859
***F3_O2**	0.8000 ± 0.1537
F3_P4	0.3416 ± 0.2167
***F3_P8**	0.5966 ± 0.2137
F3_T8	0.5500 ± 0.2838
***F3_TP8**	0.7400 ± 0.1800
F4_O1	0.2833 ± 0.1581
F4_P3	0.3583 ± 0.2153
F4_P7	0.6000 ± 0.2854
***F4_T7**	0.7750 ± 0.2153
F4_TP7	0.6166 ± 0.3047
**Intrahemispheric Pairs**	
C3_O1	0.5500 ± 0.2944
C3_P3	0.5666 ± 0.3063
C3_P7	0.8000 ± 0.2048
***C3_T7**	0.8800 ± 0.2194
C3_TP7	0.3916 ± 0.1573
C4_O2	0.6000 ± 0.2249
C4_P4	0.7000 ± 0.2452
C4_P8	0.7333 ± 0.3351
C4_T8	0.6916 ± 0.1926
C4_TP8	0.5833 ± 0.2859
F3_O1	0.2500 ± 0.1964
F3_P3	0.5666 ± 0.2629
F3_P7	0.7666 ± 0.2383
F3_T7	0.5000 ± 0.1571
F3_TP7	0.6166 ± 0.2490
F4_O2	0.3083 ± 0.1668
F4_P4	0.2166 ± 0.2086
F4_P8	0.5333 ± 0.3314
F4_T8	0.6166 ± 0.3337
F4_TP8	0.6916 ± 0.2779

∗ Mean AUC ± SD values were computed for each electrode pair using 10-fold cross-validated LASSO models. Features reflect delta- and theta-band coherence, grouped by inter- and intrahemispheric connections. Bolded features indicate LASSO-selected pairs with high discriminative power AUC $\geq$ 0.75.

Supplementary Table 3.2 THETA – AUC Values For Inter- and Intrahemispheric Pairs Across Comparisons

**Electrode Pair Comparison**	**AUC Mean ± SD**
**HC vs PD-MCI**	
**Interhemispheric Pairs**	
C3_O2	0.6777 ± 0.2960
C3_P4	0.5833 ± 0.2389
C3_P8	0.6055 ± 0.3689
C3_T8	0.2555 ± 0.2194
C3_TP8	0.6055 ± 0.3547
C4_O1	0.7011 ± 0.3280
C4_P3	0.6111 ± 0.2385
C4_P7	0.6555 ± 0.3029
C4_T7	0.5972 ± 0.2124
C4_TP7	0.5833 ± 0.2515
F3_O2	0.5777 ± 0.1892
F3_P4	0.5305 ± 0.2344
F3_P8	0.8766 ± 0.1644
***F3_T8**	0.8133 ± 0.1964
F3_TP8	0.5577 ± 0.2893
F4_O1	0.5555 ± 0.2832
F4_P3	0.6666 ± 0.2222
F4_P7	0.4777 ± 0.3366
F4_T7	0.5666 ± 0.3442
F4_TP7	0.6277 ± 0.2696
**Intrahemispheric Pairs**	
C3_O1	0.6611 ± 0.1878
C3_P3	0.6055 ± 0.2069
C3_P7	0.7000 ± 0.3220
C3_T7	0.3666 ± 0.1963
C3_TP7	0.3888 ± 0.2470
C4_O2	0.6611 ± 0.2036
C4_P4	0.5722 ± 0.1777
C4_P8	0.3222 ± 0.1938
C4_T8	0.5500 ± 0.3468
C4_TP8	0.6500 ± 0.2539
F3_O1	0.5777 ± 0.1892
F3_P3	0.6505 ± 0.2244
F3_P7	0.5166 ± 0.2144
F3_T7	0.4666 ± 0.2711
F3_TP7	0.5805 ± 0.2231
***F4_O2**	0.5888 ± 0.2774
F4_P4	0.5388 ± 0.2762
***F4_P8**	0.4777 ± 0.3366
F4_T8	0.7027 ± 0.2986
F4_TP8	0.7055 ± 0.2696
**HC vs PDD**	
**Interhemispheric Pairs**	
C3_O2	0.6583 ± 0.2790
C3_P4	0.5750 ± 0.1687
C3_P8	0.3416 ± 0.2095
C3_T8	0.4500 ± 0.1124
C3_TP8	0.7166 ± 0.2918
C4_O1	0.4750 ± 0.2888
C4_P3	0.6083 ± 0.2993
C4_P7	0.6333 ± 0.2581
C4_T7	0.6166 ± 0.3429
C4_TP7	0.6416 ± 0.2941
F3_O2	0.4750 ± 0.3558
F3_P4	0.5916 ± 0.3567
F3_P8	0.4166 ± 0.4025
***F3_T8**	0.7033 ± 0.2932
F3_TP8	0.4166 ± 0.2859
F4_O1	0.7166 ± 0.1721
F4_P3	0.4500 ± 0.3604
F4_P7	0.6000 ± 0.2108
F4_T7	0.6166 ± 0.2330
F4_TP7	0.6000 ± 0.3351
**Intrahemispheric Pairs**	
C3_O1	0.6916 ± 0.2888
C3_P3	0.6750 ± 0.2167
C3_P7	0.4333 ± 0.2509
C3_T7	0.5000 ± 0.2389
C3_TP7	0.6000 ± 0.3037
C4_O2	0.5750 ± 0.3937
C4_P4	0.3416 ± 0.1687
C4_P8	0.5125 ± 0.3298
C4_T8	0.4416 ± 0.3425
C4_TP8	0.6250 ± 0.1723
F3_O1	0.4850 ± 0.3158
F3_P3	0.2833 ± 0.2229
F3_P7	0.5583 ± 0.1844
F3_T7	0.5000 ± 0.3424
F3_TP7	0.6833 ± 0.3088
***F4_O2**	0.7116 ± 0.1798
F4_P4	0.3500 ± 0.1459
***F4_P8**	0.8350 ± 0.2034
F4_T8	0.6000 ± 0.2629
F4_TP8	0.7083 ± 0.3124
**HC vs DLB**	
**Interhemispheric Pairs**	
C3_O2	0.7750 ± 0.3425
C3_P4	0.6000 ± 0.4116
C3_P8	0.2000 ± 0.2297
C3_T8	0.7166 ± 0.2996
C3_TP8	0.6250 ± 0.4124
C4_O1	0.8500 ± 0.2687
C4_P3	0.5666 ± 0.3701
C4_P7	0.7250 ± 0.3622
C4_T7	0.5166 ± 0.4116
C4_TP7	0.7250 ± 0.3622
F3_O2	0.6416 ± 0.3748
F3_P4	0.7416 ± 0.3343
F3_P8	0.5250 ± 0.3425
***F3_T8**	0.8516 ± 0.2643
F3_TP8	0.5650 ± 0.1925
F4_O1	0.5416 ± 0.1482
F4_P3	0.6916 ± 0.3287
F4_P7	0.5916 ± 0.3736
F4_T7	0.6666 ± 0.2635
F4_TP7	0.6666 ± 0.2635
**Intrahemispheric Pairs**	
C3_O1	0.6500 ± 0.3574
C3_P3	0.2583 ± 0.2648
C3_P7	0.3833 ± 0.2918
C3_T7	0.5583 ± 0.3574
C3_TP7	0.6500 ± 0.3944
C4_O2	0.7000 ± 0.3689
C4_P4	0.3416 ± 0.2817
C4_P8	0.2583 ± 0.2058
C4_T8	0.2916 ± 0.3584
C4_TP8	0.7500 ± 0.3726
F3_O1	0.5458 ± 0.3370
F3_P3	0.5500 ± 0.3872
F3_P7	0.7166 ± 0.3220
F3_T7	0.7625 ± 0.2531
F3_TP7	0.6291 ± 0.3438
***F4_O2**	0.6766 ± 0.3488
F4_P4	0.4500 ± 0.4377
***F4_P8**	0.8966 ± 0.2495
F4_T8	0.6000 ± 0.3374
F4_TP8	0.5083 ± 0.3479
