# Supplementary material for: Wavelet event-related EEG phase coherence as a discriminant biomarker of the cognitive status in Parkinson’s and Lewy body disease
Source: Front Hum Neurosci. 2026 Apr 2;20:1696861. doi: 10.3389/fnhum.2026.1696861 (PMC13083073; doi:10.3389/fnhum.2026.1696861)
Supplement: Supplementary file 4 [file Table_4.pdf]

Supplementary Table 4. Summary of coherence–behavior and coherence–cognition regression analyses (age-adjusted).

Sample/Group	Outcome	Coherence type	β	R^2	p-value
**Delta band**					
All participants	Error score	Intrahemispheric	-0.72	0.61	< .001
		Interhemispheric	-0.61	0.47	< .01
	MMSE	Intrahemispheric	0.58	0.34	< .05
		Interhemispheric	0.53	0.30	< .05
Group-wise models					
PDD	Error score	combined	-0.82	0.67	< .001
	MMSE		0.73	0.53	< .001
PD-MCI	Error score	combined	-0.57	0.33	< .05
	MMSE		0.57	0.33	< .05
DLB	Error score	combined	-0.55	0.30	< .05
	MMSE		0.40	0.16	n.s.
**Theta band**					
All participants	Error score	Intrahemispheric	-0.53	0.46	< .05
		Interhemispheric	-0.47	0.35	< .05
	MMSE	Intrahemispheric	0.51	0.30	< .05
		Interhemispheric	0.44	0.21	< .05
Group-wise models					
PDD	Error score	combined	-0.83	0.69	< .01
	MMSE		0.71	0.51	< .01
PD-MCI	Error score	combined	-0.61	0.37	< .05
	MMSE		0.56	0.31	< .05
DLB	Error score	combined	-0.41	0.17	n.s.
	MMSE		0.33	0.11	n.s.

Note. Values represent standardized regression coefficients (β) and explained variance (R^2) from linear regression models relating event-related EEG coherence to behavioral (error score) and cognitive (MMSE) outcomes, with age included as a covariate. "All participants" models report intra- and interhemispheric coherence separately. Group-wise models were fitted within each diagnostic group as reported in the Results text. n.s. indicates non-significant.
