# Supplementary material for: Wavelet event-related EEG phase coherence as a discriminant biomarker of the cognitive status in Parkinson’s and Lewy body disease
Source: Front Hum Neurosci. 2026 Apr 2;20:1696861. doi: 10.3389/fnhum.2026.1696861 (PMC13083073; doi:10.3389/fnhum.2026.1696861)
Supplement: Supplementary file 5 [file Image_1.pdf]

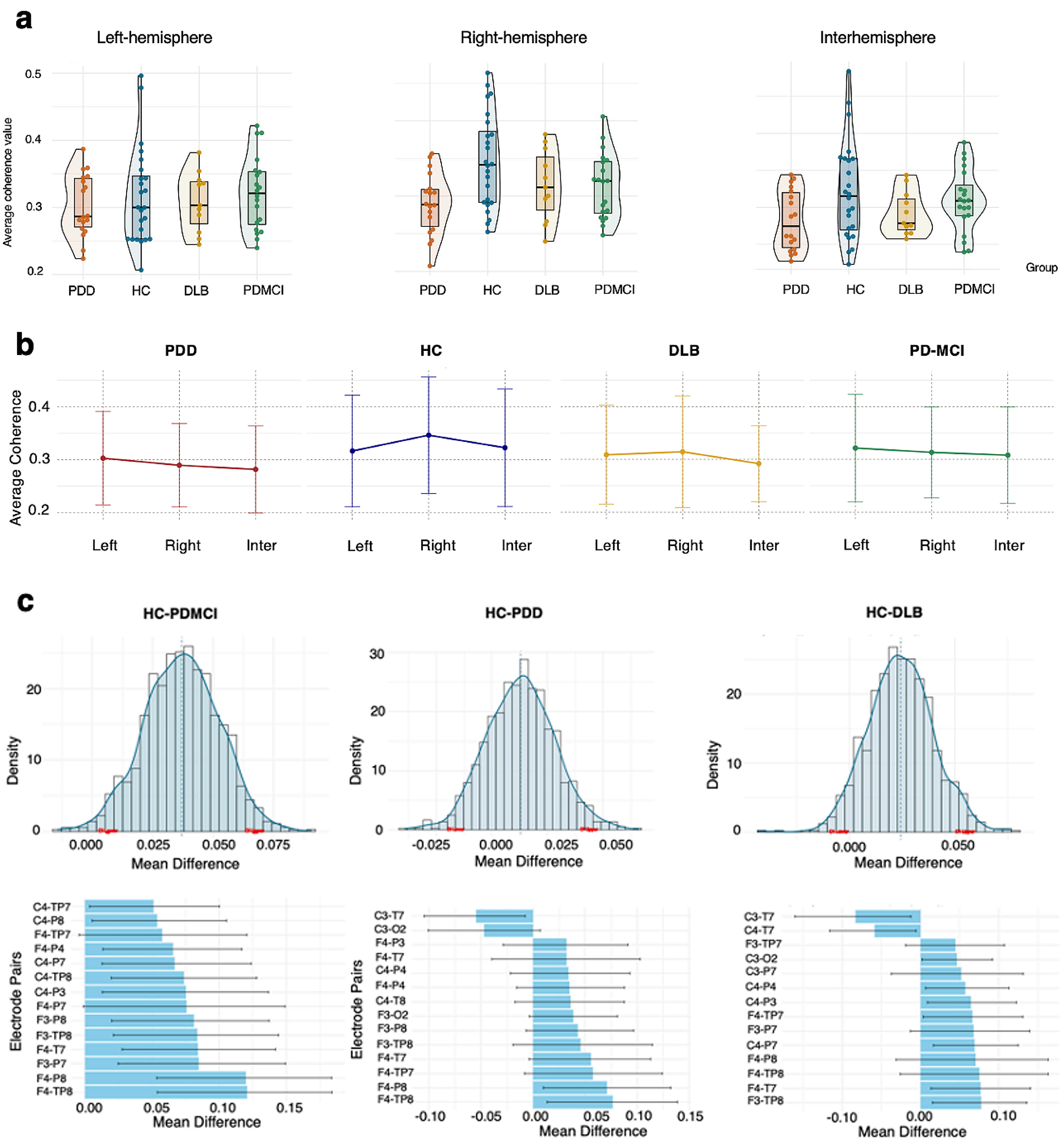

Supplementary Fig.1 | Delta-band coherence differences across groups and hemispheres. **(a)** Violin-box plots show average coherence values across left, right, and interhemispheric electrode pairs for each group (HC, PD-MCI, PDD, DLB). **(b)** ANOVA-based group means are plotted across hemispheric regions, with error bars indicating variability. **(c)** Bootstrapping and permutation analyses reveal significant group differences. Top panels show null distributions with observed mean differences (red), and bottom panels rank electrode pairs by mean difference and confidence intervals.

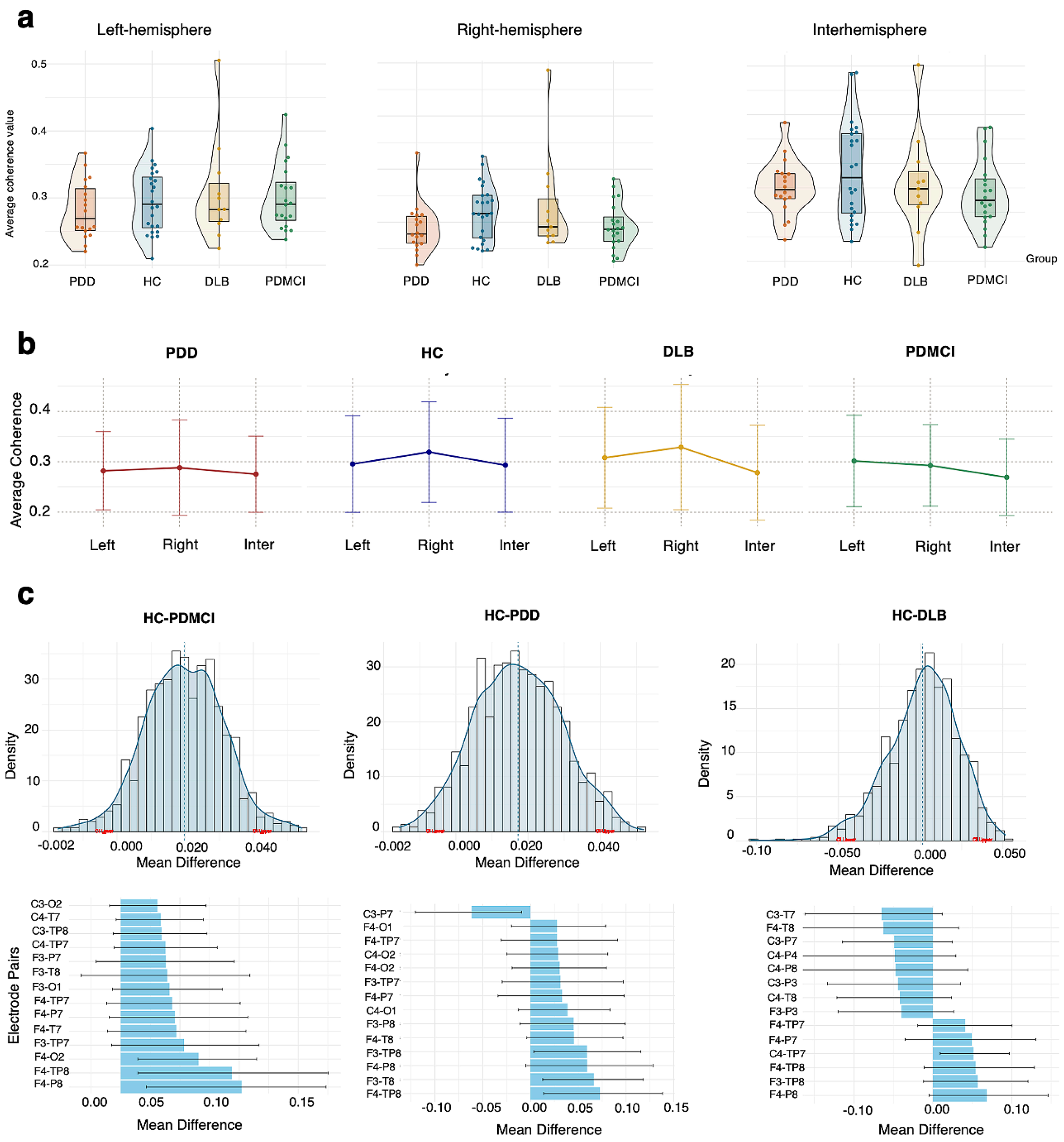

Supplementary Fig.2 | Theta-band coherence differences across groups and hemispheres. **(a)** Violin-box plots show average coherence values across left, right, and interhemispheric electrode pairs for each group (HC, PD-MCI, PDD, DLB). **(b)** ANOVA-based group means are plotted across hemispheric regions, with error bars indicating variability. **(c)** Bootstrapping and permutation analyses reveal significant group differences. Top panels show null distributions with observed mean differences (red), and bottom panels rank electrode pairs by mean difference and confidence intervals.

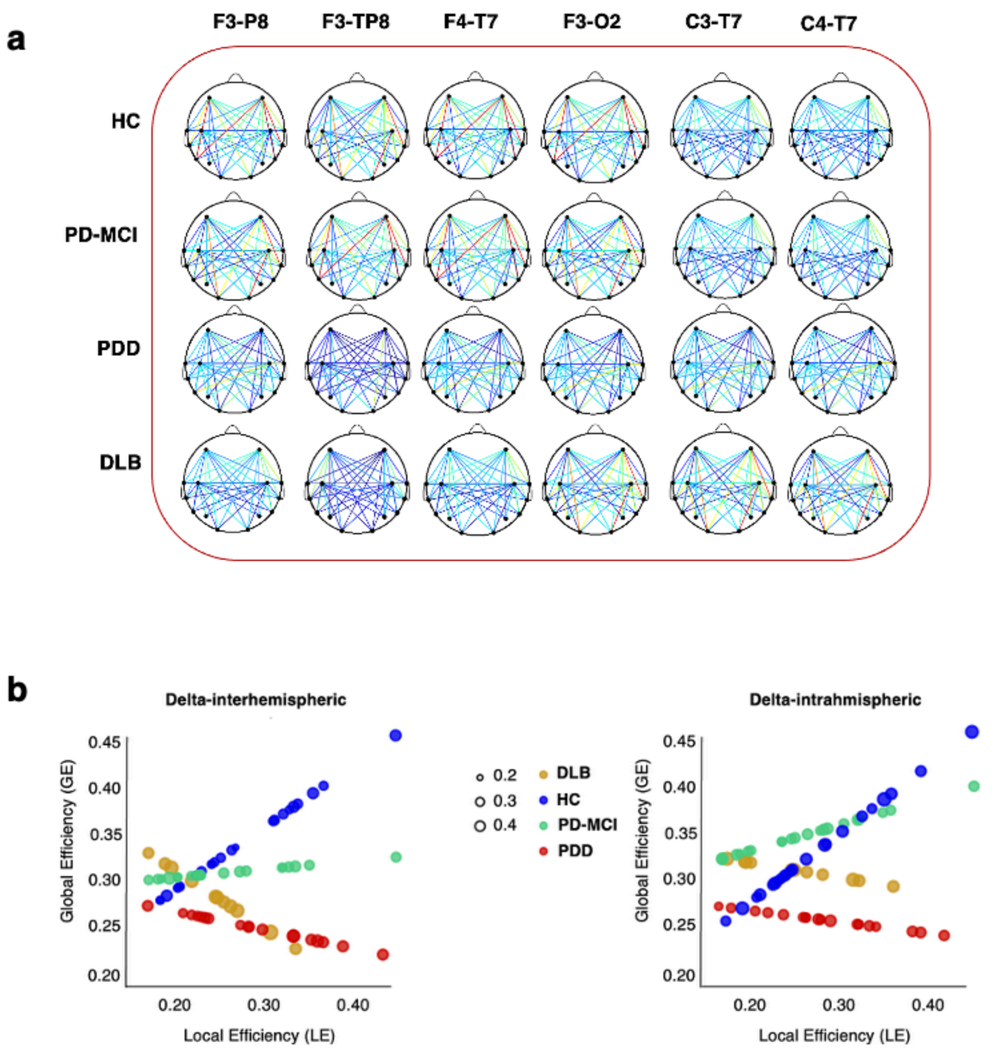

Supplementary Fig. 3 | Delta-band connectivity mapping and network efficiency. (a) Average phase coherence patterns for six LASSO-selected electrode pairs (columns), shown by group (rows). Rows represent groups (group-averaged) (HC, PD-MCI, PDD, DLB). Line color reflects coherence magnitude (blue = lower, red = higher). **(b)** Scatter plots show local efficiency (LE) versus global efficiency (GE) across interhemispheric and intrahemispheric networks; see Methods for details.

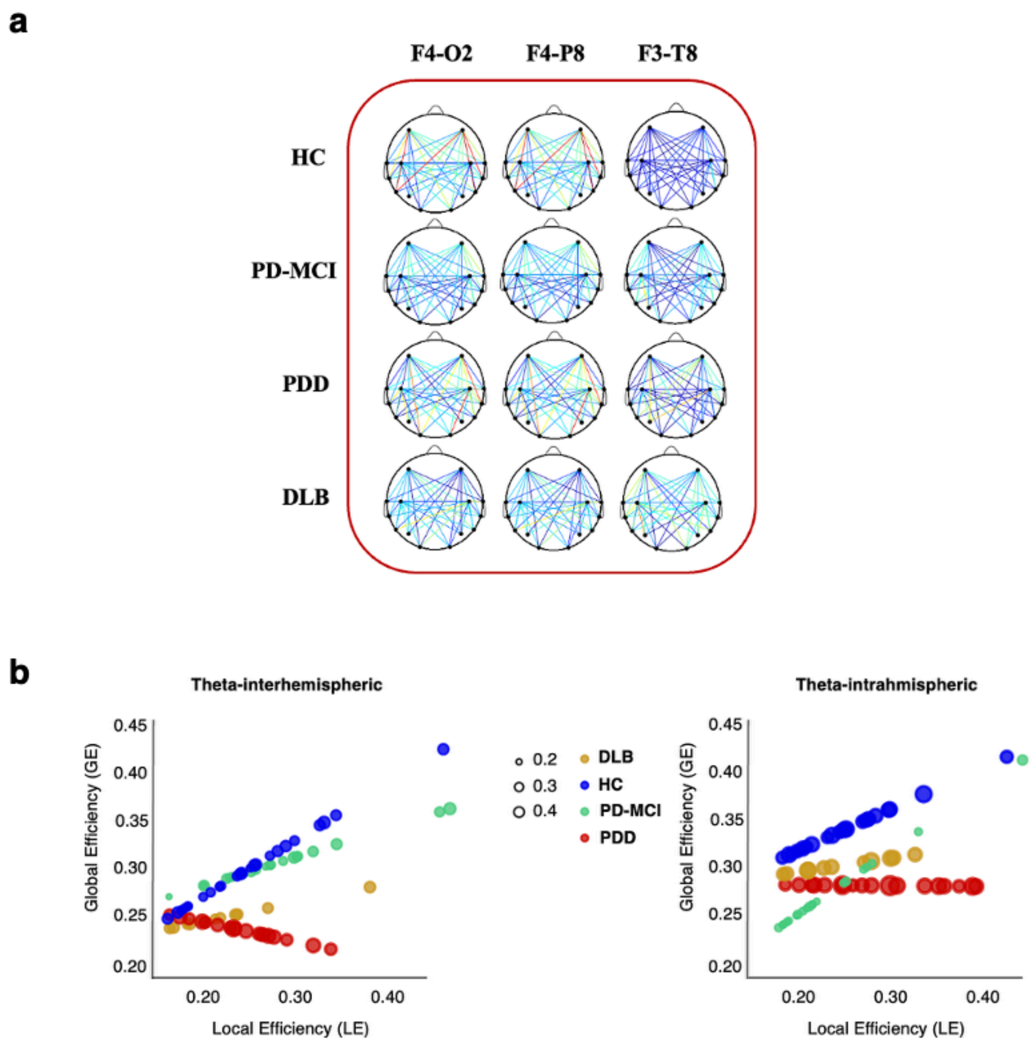

Supplementary Fig. 4 | Theta-band connectivity mapping and network efficiency. (a) Average phase coherence patterns for six LASSO-selected electrode pairs (columns), shown by group (rows). Rows represent groups (group-averaged) (HC, PD-MCI, PDD, DLB). Line color reflects coherence magnitude (blue = lower, red = higher). **(b)** Scatter plots show local efficiency (LE) versus global efficiency (GE) across interhemispheric and intrahemispheric networks; see Methods for details.

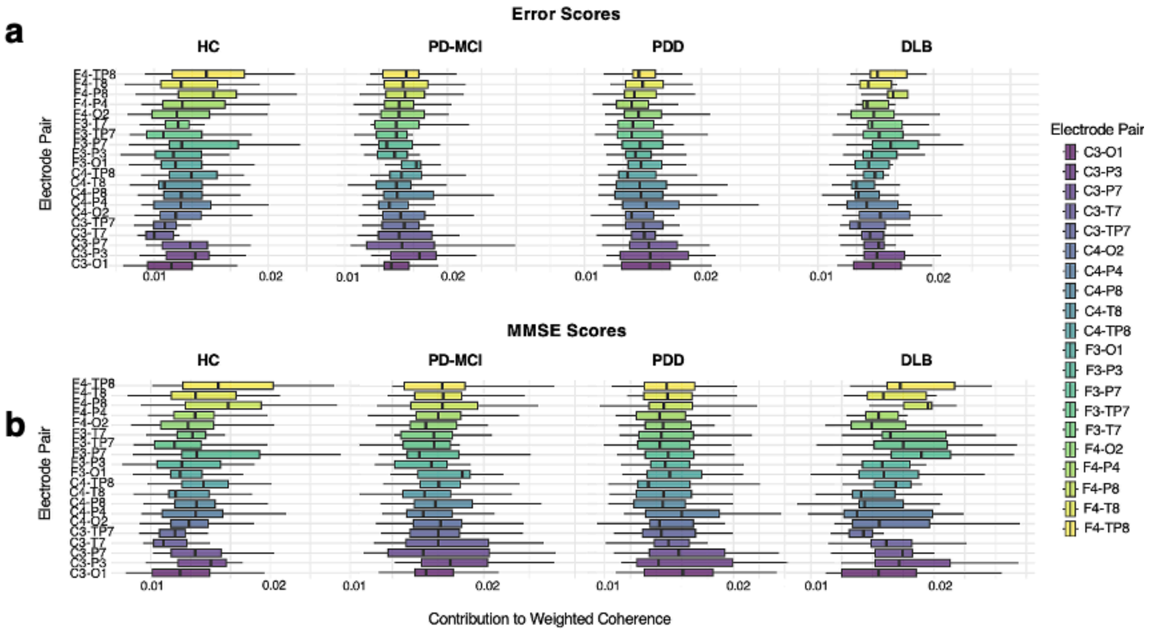

(Interhemispheric Pairs)

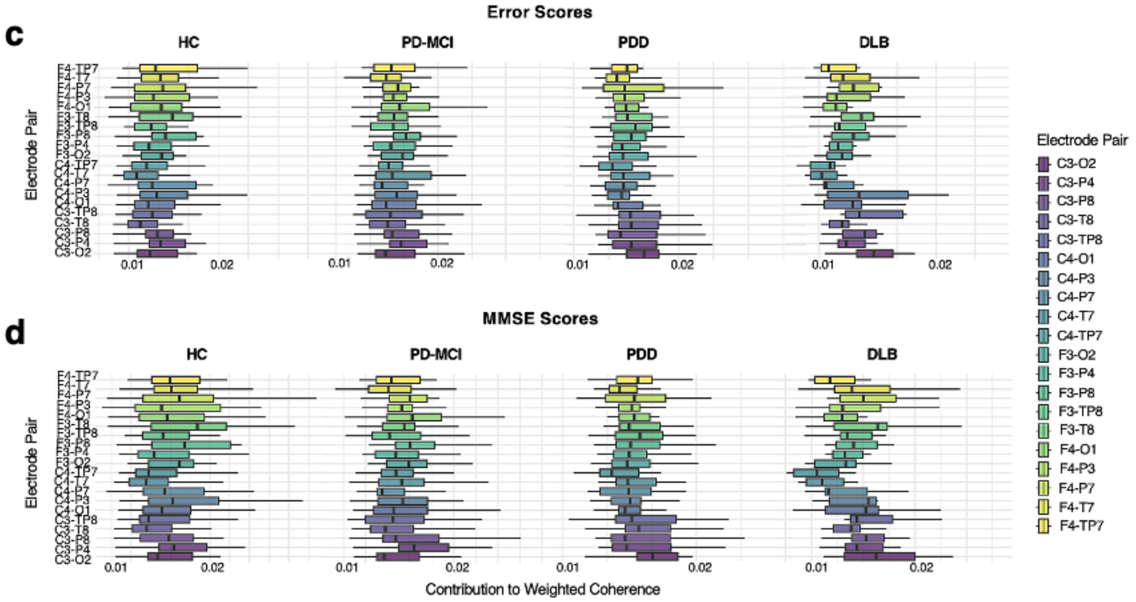

Supplementary Fig.5 | Delta Band Coherence – Predictive contributions of coherence features to behavioral and cognitive scores. Bars show average normalized contribution of each coherence feature (weighted) to error score and MMSE score prediction across groups via regression. **(a–b)** Intrahemispheric; **(c–d)** interhemispheric pairs.

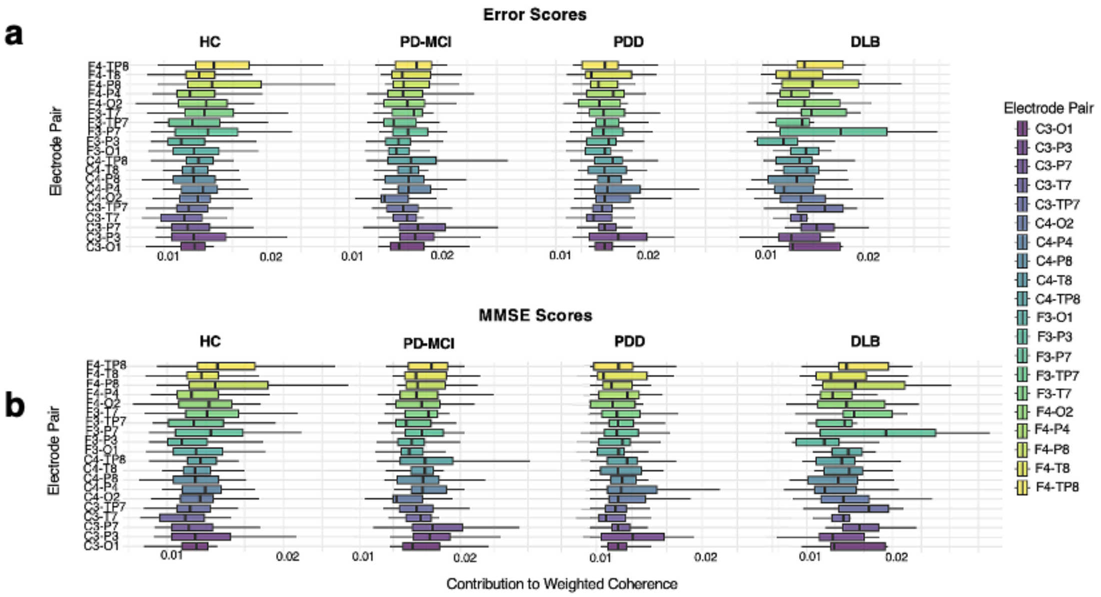

(Interhemispheric Pairs)

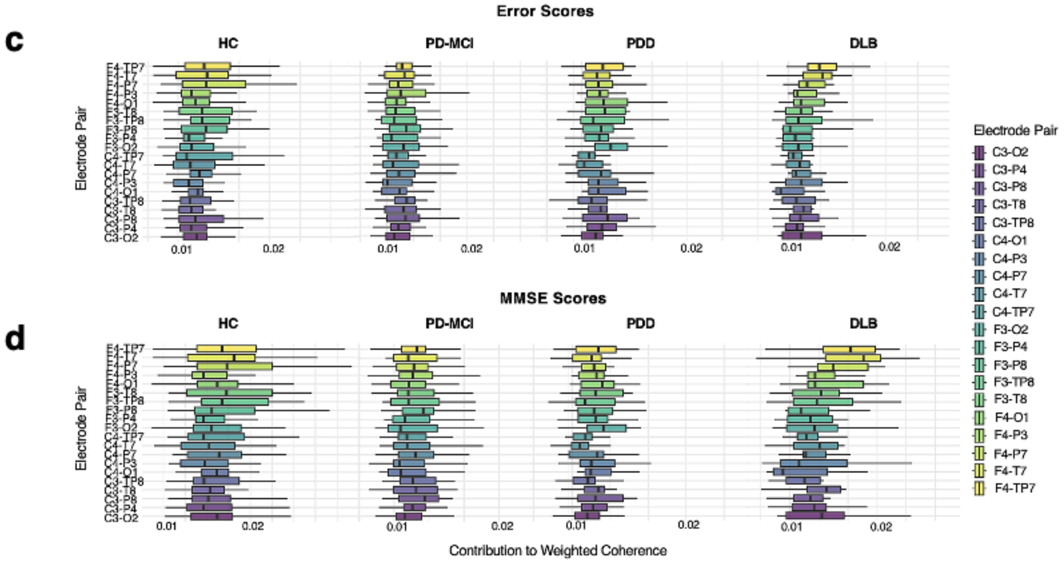

Supplementary Fig.6 | Theta Band Coherence – Predictive contributions of coherence features to behavioral and cognitive scores. Bars show average normalized contribution of each coherence feature (weighted) to error score and MMSE score prediction across groups via regression. **(a–b)** Intrahemispheric; **(c–d)** interhemispheric pairs.
